# Label-free nonlinear optical signatures of extracellular vesicles in liquid and tissue biopsies of human breast cancer

**DOI:** 10.1038/s41598-024-55781-4

**Published:** 2024-03-06

**Authors:** Janet E. Sorrells, Jaena Park, Edita Aksamitiene, Marina Marjanovic, Elisabeth M. Martin, Eric J. Chaney, Anna M. Higham, Kimberly A. Cradock, Zheng G. Liu, Stephen A. Boppart

**Affiliations:** 1 Beckman Institute for Advanced Science and Technology, University of Illinois Urbana-Champaign, Urbana, IL 61801 USA; 2 Department of Bioengineering, University of Illinois Urbana-Champaign, Urbana, IL 61801 USA; 3 NIH/NIBIB P41 Center for Label-Free Imaging and Multiscale Biophotonics (CLIMB), University of Illinois Urbana-Champaign, Urbana, IL 61801 USA; 4 Cancer Center at Illinois, Urbana, IL 61801 USA; 5 Carle Foundation Hospital, Urbana, IL 61801 USA; 6 Department of Electrical and Computer Engineering, University of Illinois Urbana-Champaign, Urbana, IL 61801 USA; 7 Interdisciplinary Health Sciences Institute, University of Illinois Urbana-Champaign, Urbana, IL 61801 USA

**Keywords:** Diagnostic markers, Breast cancer, Multiphoton microscopy

## Abstract

Extracellular vesicles (EVs) have been implicated in metastasis and proposed as cancer biomarkers. However, heterogeneity and small size makes assessments of EVs challenging. Often, EVs are isolated from biofluids, losing spatial and temporal context and thus lacking the ability to access EVs in situ in their native microenvironment. This work examines the capabilities of label-free nonlinear optical microscopy to extract biochemical optical metrics of EVs in ex vivo tissue and EVs isolated from biofluids in cases of human breast cancer, comparing these metrics within and between EV sources. Before surgery, fresh urine and blood serum samples were obtained from human participants scheduled for breast tumor surgery (24 malignant, 6 benign) or healthy participants scheduled for breast reduction surgery (4 control). EVs were directly imaged both in intact ex vivo tissue that was removed during surgery and in samples isolated from biofluids by differential ultracentrifugation. Isolated EVs and freshly excised ex vivo breast tissue samples were imaged with custom nonlinear optical microscopes to extract single-EV optical metabolic signatures of NAD(P)H and FAD autofluorescence. Optical metrics were significantly altered in cases of malignant breast cancer in biofluid-derived EVs and intact tissue EVs compared to control samples. Specifically, urinary isolated EVs showed elevated NAD(P)H fluorescence lifetime in cases of malignant cancer, serum-derived isolated EVs showed decreased optical redox ratio in stage II cancer, but not earlier stages, and ex vivo breast tissue showed an elevated number of EVs in cases of malignant cancer. Results further indicated significant differences in the measured optical metabolic signature based on EV source (urine, serum and tissue) within individuals.

## Introduction

Extracellular vesicles (EVs) are nanometer- to micrometer-sized bilipid-membrane bound particles released from all cell types^1^. EVs play a role in cell-to-cell communication and can transport encapsulated molecules between organs via the circulatory system. In cancer, EVs have been implicated for their role in metastasis^2^ and in promoting tumorigenesis in the local tumor environment through processes such as immunosuppression^3,4^ and angiogenesis^5,6^. Furthermore, breast cancer EVs can reprogram the metabolism of target cells^7^. Despite these findings, there are still many unknowns regarding the systemic roles that EVs play in cancer and metastasis due to the difficulty of studying them in vivo and in diverse environments.

EVs can be isolated from easily accessible biofluids such as blood serum and urine, and have been suggested as candidates in “liquid biopsy” for screening and diagnostics for a variety of different diseases including cancer^8^. These biofluids are collected for routine tests in all medical care facilities, which would enable convenient translation of biofluid-derived EV tests into current standard-of-care. One significant barrier to characterizing EVs in liquid biopsy is the lack of single-EV analysis methods with biochemical/functional specificity. Most methods to analyze EVs focus on “bulk” analysis that provides indiscriminatory genomic, metabolomic, or proteomic information for a sample with a large number of EVs, or rely upon structural or physical features such as using nanoparticle tracking analysis (NTA) for EV size and concentration^9^, dynamic light scattering (DLS) for EV size distribution^10^, transmission electron microscopy (TEM) for EV size and morphology^11^, or atomic force microscopy for EV size and physical properties^12^. Single-EV analysis methods are needed to untangle the heterogeneity of EVs, especially in early cancer detection where only a small proportion of EVs may be indicative of disease^13^. In recent years, several studies achieved label-free biochemical characterization of EVs using methods such as spontaneous Raman spectroscopy^14,15^ and surface-enhanced Raman spectroscopy^16–18^. Raman methods can achieve single-EV resolution and have discovered high biochemical heterogeneity in EVs^19^. Flow cytometry can additionally be used for single-EV level resolution to examine surface markers^20^. Single-EV proteomic analysis has recently been achieved via droplet barcode sequencing^21^. However, these methods lack the ability to characterize EVs in situ, either in vivo or ex vivo, which provides essential spatial and dynamic information. In vivo imaging of EVs with exogenous fluorescent markers presents a pathway to EV tracking and visualization in situ^22,23^, but these methods lack information on the functional state and biochemical makeup of EVs.

Single-EV functional and biochemical characterization of both isolated and in situ EVs was recently achieved with label-free nonlinear optical microscopy using endogenous contrasts from autofluorescent molecules within EVs^24–30^. Autofluorescence is primarily collected from reduced nicotinamide adenine dinucleotide (phosphate) (NAD(P)H) and flavin adenine dinucleotide (FAD), key metabolic cofactors that are ubiquitous throughout biological systems. The relative autofluorescence intensities and lifetimes of these cofactors are dependent on the activity of metabolic pathways within cells, and these cofactors are passed on into EVs. Optical metrics quantifying NAD(P)H and FAD can thus be used to make inferences regarding cells and the EVs they produce. The autofluorescence of these cofactors has been studied extensively in cell culture^15,31,32^ and tissues^33,34^ in a variety of healthy and disease states including cancer. Importantly, due to the cancer-related shift in energy metabolism toward reliance on glycolysis instead of mitochondrial metabolism, NAD(P)H and FAD autofluorescence show distinct signatures in cancer^31,33,34^ and in other altered metabolic states^15,35^. This is attributed to the difference in relative abundance of NAD(P)H and FAD as metabolic pathways shift and the longer fluorescence lifetime of protein-bound NAD(P)H compared to “free” NAD(P)H^36–39^.

Label-free nonlinear optical microscopy can quantify and spatially map these shifts in relative abundance via intensity-based imaging and shifts in the protein-bound vs. free states with fluorescence lifetime imaging microscopy (FLIM). One label-free nonlinear optical microscopy method previously used to characterize EVs is simultaneous label-free autofluorescence multiharmonic (SLAM) microscopy^40^. Briefly, this method uses a spectrally- and temporally-optimized pulsed laser excitation to simultaneously image multiple nonlinear optical contrast mechanisms within biological samples: two-photon excited autofluorescence of FAD, three-photon excited autofluorescence of NAD(P)H, second harmonic generation (SHG) of collagen, and third harmonic generation (THG) of interfaces to visualize sample structure. From SLAM images, the optical redox ratio (ORR), a normalized metric to characterize the redox balance, can be computed from FAD and NAD(P)H intensities. From multiphoton FLIM of NAD(P)H, the mean fluorescence lifetime (τ), and phasor components (g and s) are computed to describe the multidimensional fluorescence decay curve. EVs contain active enzymes^41^, which suggests that the protein-bound NAD(P)H signature in EVs is due to enzymatic activity of proteins that use NADH or NADPH as cofactors (NADH and NADPH exhibit similar fluorescence excitation and emission, and their optical signature is often referred to as NAD(P)H to account for the contribution from both molecules). Thus, the fluorescence lifetime provides a functional quantification of what is occurring inside the EV, and may provide insight on the parent cell metabolism.

Label-free microscopy has previously been used to explore the optical signatures of isolated EVs from cell culture media^24,25^, ex vivo EVs in breast cancer tissue^24,26,27^, in vivo EVs in rat mammary tumor tissue^24^ and human patient-derived xenografts from pancreatic cancer implanted into mice^28^, isolated urinary EVs in canine bladder cancer^29^, and isolated urinary EVs in human colorectal cancer^30^. These studies have indicated that the optical characteristics of EVs are strongly associated with cancer. Notably, one study showed that in vivo rat tumors exhibited a significantly elevated concentration of NAD(P)H-rich EVs^24^, a biomarker that is indicative of the metabolic preference of cancer cells for glycolysis, which leads to increased NADH levels. Previous work also showed that EVs isolated from cell culture had significantly different NAD(P)H fluorescence lifetime than parent cells, but with a lifetime profile most similar to that of the parent cell cytosol, and less similar to the parent cell nucleus and mitochondria^25^, indicating that EVs can be representative markers of parent cell metabolism. These previous studies using label-free nonlinear optical microscopy have primarily focused on large EVs. Likely due to their larger volume which enables larger EVs to carry a higher number of autofluorescent molecules, large EVs produce a stronger autofluorescence signal than small EVs^25^. In this manuscript “EV” is used as a general term to refer to both isolated large EVs (pelleted by ultracentrifugation at 12,000×*g*) and non-isolated EVs in tissue.

Here we explore and compare the optical metabolic signatures of isolated EVs from urine and serum with in situ EVs in freshly excised intact ex vivo breast tissue from human study participants. We explore and compare optical signatures between groups of healthy and breast cancer participants, including benign and malignant breast cancer from stages 0 to II. This work presents the first inter- and intra-individual comparison of optical metabolic signatures across multiple EV sources. Furthermore, it presents the first exploration into the capability of using optical metabolic signatures of EVs in liquid biopsy for breast cancer. A limited number of participants (n = 34) were recruited in this preliminary study in order to understand capabilities and establish candidate biomarker signatures for future, larger-scale analyses. Results indicate that several optical metabolic metrics have the potential to indicate the presence of early-stage cancer and that optical metabolic signatures differ greatly across EV sources.

## Methods

### Ethics statement

This study involved human participants and was approved by Carle Foundation Hospital and University of Illinois Urbana-Champaign (Urbana, IL) Institutional Review Boards (IRB #18CCC1708). All research procedures performed were in accordance with the ethical standards of the Institutional and National research committee and with the 1964 Helsinki Declaration and its later amendments or comparable ethical standards. Written informed consent was obtained from all individual participants involved in the study and all eligible participants completed a one-time medical history questionnaire. All confidential data was stored a in REDCap (Research Electronic Data Capture) database.

### Participant selection

**Figure 1:**
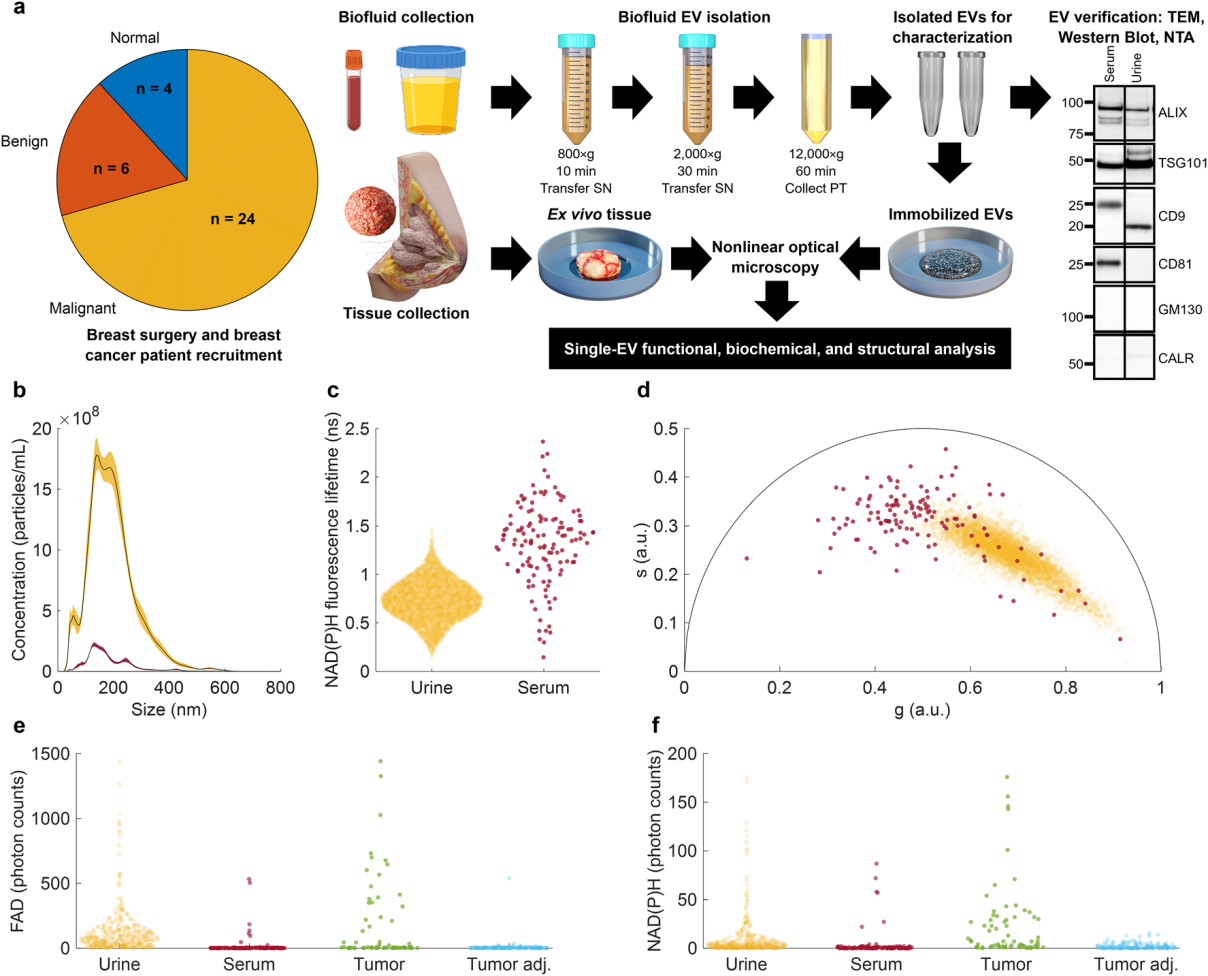
Workflow for characterizing EVs in breast cancer using single-EV capabilities of nonlinear optical microscopy and example dataset from one participant. (**a**) Study overview including participant cancer status, EV isolation, and tissue extraction, and imaging. (**b**) Concentration and size of isolated EVs. (**c**) NAD(P)H single-EV fluorescence lifetime distribution; (**d**) NAD(P)H single-EV phasor distribution for isolated EVs from urine (yellow) and serum (red) from a single participant with malignant breast cancer. (**e**) Single-EV FAD autofluorescence intensity distribution. (**f**) Single-EV NAD(P)H intensity distribution for isolated EVs from urine (yellow), serum (red), ex vivo tumor tissue (green), and normal-appearing ex vivo tissue from the same participant “Tumor adj.” (light blue). Panels (**b**–**f**) all show data collected from samples from one single participant with malignant breast cancer, indicating the broad range of different variables that were collected from each participant.

The study accrued 40 participants in total: 30 malignant breast cancer patients, 6 benign breast tumor patients, and 4 individuals without cancer scheduled for breast reduction surgery as normal controls. Not all samples were able to be processed and imaged due to logistical considerations; only samples able to be processed and successfully imaged are presented, leading to a final cohort of 34 participants: 24 breast cancer-positive, 6 benign breast tumor, and 4 breast-reduction surgery normal controls (Fig. 1a). Serum and urine were collected from all participants, and ex vivo tissue was collected from participants when available (tumor and/or extra non-tumor tissue > 1 cm in size). Participant demographics and relevant health information (including age, race/ethnicity, clinical staging of breast cancer, and more) collected from the electronic medical record (EMR) is provided in Table S1, as well as which biofluid and tissue samples were available and imaged from each participant. Cancer stages of malignant tumors and patient age are graphically represented in Fig. S1.

### Tissue specimen collection

Human breast tissue specimens were collected during either breast-conserving surgery (lumpectomy) or mastectomy, for malignant or benign tissues, or during breast reduction surgery (normal tissue, no patient history of cancer). The resected specimens were placed in a phosphate buffered saline (PBS) and the cold ischemia start time was documented. After no more than 45 min past cold ischemia time, specimens were bread-loafed to identify the gross tumor (if present). If the tissue was > 1 cm in size, the pathologist or assistant selected a small sample of tissue from the tumor and/or visually healthy tissue and placed the sample(s) in vials with saline. Visually healthy tissue from participants with malignant breast cancer was termed “tumor adjacent tissue” whereas visually healthy tissue from breast reduction surgery participants was considered to be a healthy control. In some cases, only tumor tissue or tumor adjacent tissue was available from a patient with malignant cancer (Table S1). Tumor stage and grade were determined by a board-certified pathologist. Tissue samples were transported to the imaging lab on ice and imaged within 2–3 h of excision.

### Biofluid collection

Pre-operative urine samples were collected from the research participants in 120 mL sterile urine specimen cups at a random time of day. Pre-operative whole venous blood was collected from research participants into 10 mL BD Vacutainer polyethylene terephthalate serum separation tubes (SST). Urine and blood samples were transported to the sample processing laboratory on ice within 1 h of collection and processed within 1 h after receiving. Sample volumes are given in Table S2.

### EV isolation

The workflow from sample retrieval to EV imaging, including EV isolation and validation, is presented in Fig. 1a. EVs were isolated from biofluids as previously described^29^. Urine samples were diluted to a final volume of 45 mL using PBS if less than 45 mL was acquired. If more than 45 mL was acquired, only 45 mL was used and the remaining sample was discarded. After clotting for 30 min, the venous whole blood was centrifuged at 1200×*g* for 10 min to separate serum from red blood cells. A 3–5 mL volume of serum was collected and resuspended in a final 42 mL PBS volume. Both serum and urine samples were centrifuged at 800×*g* for 10 min at 4 °C. A 40 mL volume of supernatant was transferred into new 50 mL conical Protein LoBind tube (Eppendorf), which was centrifuged at 2000×*g* for 30 min at 4 °C to remove particulate matter. These first two centrifugation steps were performed in a benchtop centrifuge (Rotina 380 R, Hettich). Supernatant (34 mL) was centrifuged at 12,000×*g* for 1 h at 4 °C to isolate EVs in an ultracentrifuge (Sorvall WX +, Thermo Fisher) using a Surespin630/36 rotor and associated tubes (PP thinwall tube, 36 mL, Thermo Scientific). The resulting urinary EV or serum EV pellet was dissolved in 55 μL of PBS supplemented with 25 mM D(+)-Trehalose (Fig. 1a). This type of procedure is sometimes referred to as isolating large EVs (LEVs) or microvesicles (MVs); throughout the manuscript we use the general term “EV” and indicate whether that EV sample was isolated from biofluids using this procedure or whether EVs were segmented from images of intact tissue.

### EV counting and size distribution analysis

Isolated EV count and size were evaluated by NTA (NanoSight NS300, Malvern Panalytical, Malvern, UK). The EV analytes were diluted in PBS to a final volume of 2 mL. In NTA, the dilution factor was recorded during each run to enable an automated particle concentration upgrade by the NTA v.3.3 (104) software. The data was primarily acquired using a camera sensitivity level set to either 12 or 13 and syringe pump speed set to 75. Valid particle track number was determined at a detection threshold of 3 or 5. Each NTA run lasted for 3 min. Video data capture time was set to either 30 s or 60 s to collect 6 or 3 repeated measurement data points. The main NTA results were EV concentration (expressed as particles/mL normalized to the initial volume of raw sample) and mean diameter (Fig. 1b).

### Transmission electron microscopy

The presence of EVs in the samples that were processed by differential centrifugation was confirmed by transmission electron microscopy (Fig. S3). For TEM imaging, 20 μL of EVs were adsorbed to a 200-mesh Formvar-coated copper grid (Ted Pella) for 3 min. Excess sample was drained using filter paper before being stained negatively with a 2% w/v uranyl acetate solution. The samples were examined by TEM at high magnification (FEI Tecnai G2 F20 S-TWIN STEM, 120 kV).

### Western blotting

The expression of EV biomarkers was detected by Western blotting, which was performed as described previously^42^. Proteins from lysed serum or urine EVs were separated by LDS-PAGE under reducing conditions using 4–12% gradient NuPAGE gels. Resolved proteins were transferred onto 0.22 µm pore-size nitrocellulose membrane, which was subdivided into distinct molecular weight zones, blocked with 3% BSA solution for 2 h and individually immunoblotted with a 1:1000 dilution of either unconjugated Anti-ALIX rabbit monoclonal antibody (Boster-Bio, #M01751), anti-TSG101 rabbit polyclonal antibody (Abcam, #ab30871), anti-CD9 rabbit monoclonal antibody (Boster-Bio, #M01202), anti-CD81 rabbit monoclonal antibody (Boster-Bio, #M01281-1), Anti-GM130/GOLGA2 (Golgi organelle contamination indicator) mouse monoclonal 6D4 antibody (Boster-Bio, # M05865-2) or anti-Calreticulin/CALR (ER organelle presence indicator) (Boster-Bio, #A00894-1) antibody overnight followed by four 7 min wash cycles in prechilled 1X TBS-T buffer, probing with 1:10,000 horse anti-mouse IgG Cell Signaling Technology, #7076S) or 1:40,000 goat anti-rabbit IgG (H + L) (Thermo Fisher, #31460) HRP-linked secondary antibody for 1 h at room temperature, four 5 min washes in 1X TBS-T buffer and a final wash in dH2O prior to chemiluminescent signal development by a 5 min-long blot incubation with SuperSignal™ West Pico PLUS chemiluminescent substrate (Thermo Scientific) and automated protein band visualization in Bio-Rad ChemiDoc MP imaging system at optimal non-saturating 24 s (ALIX), 44 s (TSG101), 180 s (CD9, CD81, GM130 and CALR) exposure time. Data is shown in Fig. 1a.

### EV verification and validation

The minimal information for studies of extracellular vesicles 2018 (MISEV2018) guidelines provide an extensive set of parameters to report and control in EV-related studies^43^. Detailed steps for EV isolation via ultracentrifugation are described above and initial sample volumes are given in Table S2, as suggested by MISEV2018. For EV validation, NTA, TEM, and Western blotting were used, as described above; the use of multiple complementary techniques is strongly suggested by MISEV 2018. Using Western blotting, ESCRT components ALIX and TSG101 were confirmed in isolated EVs, along with tetraspanins CD9 and CD81. GM130 and CALR are associated with non-EV intracellular components, were also examined. Accordingly, the term “extracellular vesicle” or “EV” was used as a general term since general EV size, morphology, and markers were confirmed in the isolated particles examined in this study, but no specific subtype of EV was selected with certainty.

### Nonlinear optical microscopy

EVs were imaged on two different previously described custom nonlinear optical microscopy systems, both configured in an inverted epi-detection geometry using 1.05 NA water immersion objective lenses (XLPLN25XWMP2, Olympus)^24,25^. While detailed system diagrams and descriptions are available in previous publications^24,25^, both imaging systems are depicted in Fig. S2. For imaging with SLAM microscopy, isolated EVs were embedded in 99% molecular biology grade glycerol (Sigma, USA) in a 1:4 EVs:glycerol ratio^24,29^. Fresh ex vivo breast tissue specimens were also imaged on the SLAM system. Samples were kept on ice until imaging, and then placed on top of a large cover glass (260454, Ted Pella) and imaged at room temperature from below. Briefly, the SLAM system uses a shaped supercontinuum (1110 ± 30 nm, 35 fs) to excite four different types of nonlinear optical contrast within biological specimens: SHG, THG, two-photon excited autofluorescence of FAD, and three-photon excited autofluorescence of NAD(P)H^26,40^. Thus, each pixel of a SLAM image contains four channels of intensity, each corresponding to one of these four contrasts (Fig. S2a). SHG primarily shows collagen in biological samples, and is not present in EVs. THG derives contrast from changes in refractive index, highlighting biological features such as cell and EV membranes. Samples were imaged using 14 mW of incident power, with a 200 × 200 μm field of view (FOV), with a frame rate of 0.125 Hz, and no frame averaging. For biofluid-derived EVs, 25 total FOV per sample were acquired; for tissue samples, 9–81 total FOV were acquired per sample depending on sample size.

EVs were additionally characterized with two-photon exited FLIM to characterize NAD(P)H-protein interactions^25^. This custom system creates a time-trace for each pixel by time-tagging collected photons in order to characterize the fluorescence decay curve of each pixel of the image (Fig. S2b). From this curve, fluorescence lifetime and associated metrics can be determined, enabling inferences about the free and protein-bound NAD(P)H within the sample. For FLIM, EVs were embedded in a 0.2% agarose gel in a 1:5 ratio (EVs in PBS:agarose) and imaged with an average power of 25 mW using 750 nm excitation. An average of 20 frames per FOV was acquired, with 16 total FOV per sample, each 180 × 180 μm. The frame rate of the system was 0.2 Hz. The number of EVs imaged per sample is given in Table S3; if 10 or fewer EVs were imaged, the dataset was discarded and excluded due to low number. The mean number of EVs imaged per sample were: 1330 for urinary EV FLIM, 579 for urinary EV SLAM, 134 for serum EV FLIM, 691 for serum EV SLAM, 26,954 for ex vivo tumor SLAM, and 8,228 for ex vivo non-tumor SLAM, with large standard deviations similar to or larger than the means, as listed in Table S3.

### Image processing, interpretation, and statistical analysis

Image/data analysis and visualization was performed using custom Matlab scripts. EVs were segmented using a blob-detection algorithm, based on previously demonstrated^25,28^ and validated methods^24,27^, but segmentation validation using tagged EVs was not performed in this study due to the limited number of EVs per sample and potential for fluorescence bleedthrough. Mean fluorescence lifetime (τ) was determined using phasor analysis to determine the cosine (g) and sine (s) components at the modulation frequency of the laser (ω)^44^; ORR was computed the same as in previous EV studies^24,29^.

1 $$\tau \, = {\text{s}}/\left( {\omega {\text{g}}} \right)$$

2 $${\text{ORR }} = {\text{FAD}}/\left( {{\text{FAD }} + {\text{ NAD}}\left( {\text{P}} \right){\text{H}}} \right)$$

The mean fluorescence lifetime (τ) of NAD(P)H increases when it is bound to a protein; an increase is associated with more NAD(P)H-related protein activity in the EVs. Phasor components g and s represent the fluorescence lifetime profile in a two-dimensional space that can be used to better represent and understand combinations of multiple lifetime species within one pixel, such as combinations of free and protein-bound NAD(P)H. Phasor components are useful since they can describe a shift in the fluorescence decay curve that is not evident in just the mean lifetime, since the mean lifetime is made of multiple components of bound and free NAD(P)H. ORR is generally assumed to correspond to an increase in mitochondrial activity, where FADH_2_ is converted to FAD (leading to an increase in FAD autofluorescence signal) and NADH is converted to NAD + (leading to a decrease in NADH autofluorescence signal). Thus, high ORR is associated with relatively higher mitochondrial activity and lower glycolytic metabolism (in which NAD + is reduced to NADH, increasing NADH autofluorescence signal), and low ORR is associated with relatively lower mitochondrial activity and higher glycolytic metabolism. It is important to note that multiple formulae for ORR and related metrics exist, which should be carefully considered when comparing these results to those of other studies.

To compare matched data from the same participant (ex: mean FAD in urine vs. serum), paired Student’s t-tests were used. To compare data from different participants (ex: mean FAD in malignant vs. benign tumors), non-paired Student’s t-tests were used. Linear correlation coefficient (R) was additionally calculated to compare optical metabolic signatures across participants, and additionally used to examine confounding variables such as participant age.

## Results and discussion

### Label-free nonlinear optical microscopy enables multidimensional analysis with single-EV resolution

**Figure 2:**
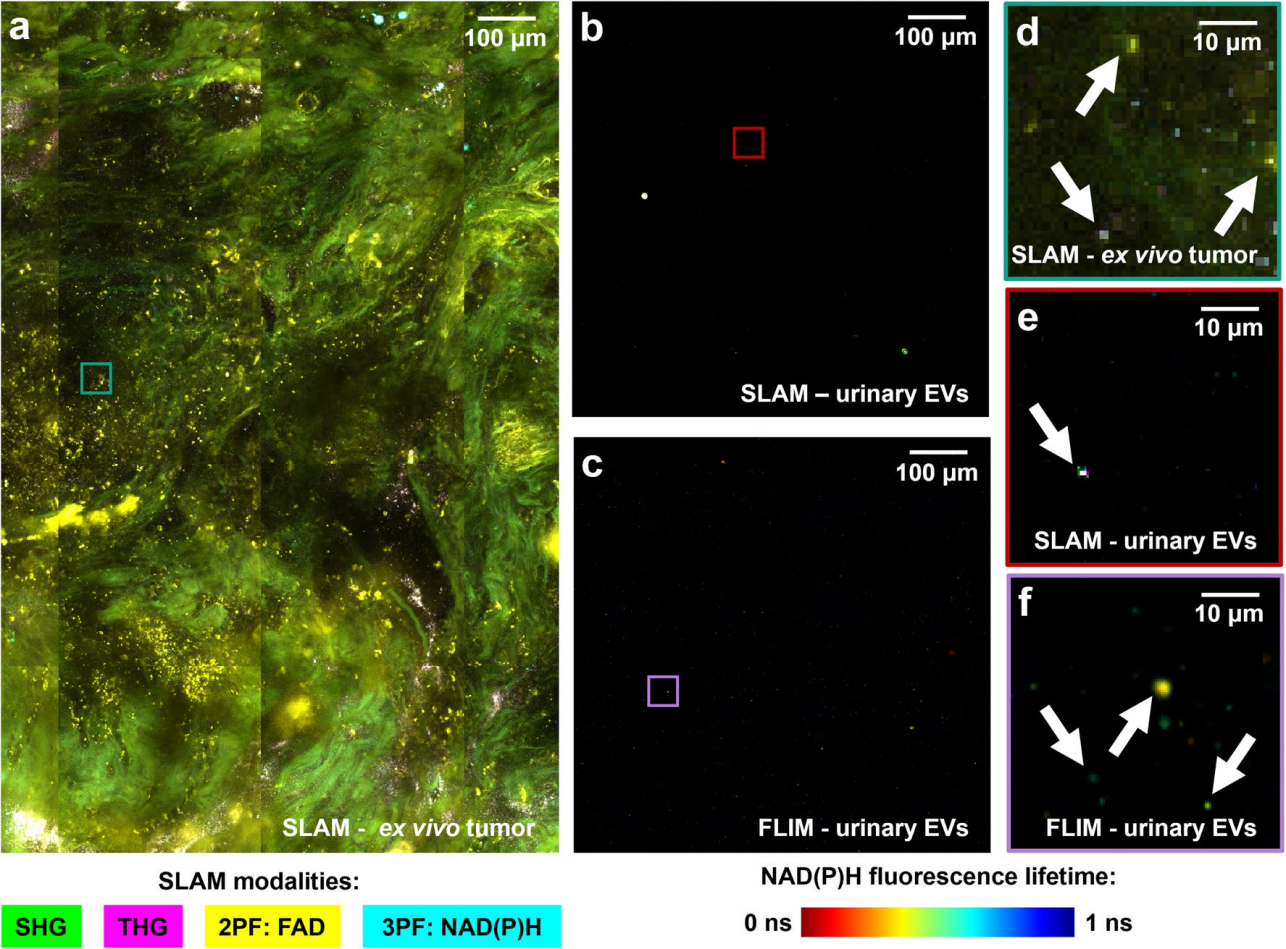
Example images of EVs in situ in tissue and isolated EVs. (**a**) Example SLAM image of ex vivo tumor tissue with 4-color map displaying each SLAM modality. (**b**) Example SLAM image of urinary isolated EVs. (**c**) Example FLIM image of urinary isolated EVs with rainbow colormap displaying mean NAD(P)H fluorescence lifetime. (**d**–**f**) Insets corresponding to smaller field of view from (**a**–**c**), respectively, showing single-EVs zoomed-in.

Intact breast tissue and biofluid-derived isolated EVs from urine and serum were imaged with label-free nonlinear optical microscopy to characterize and extract the optical metabolic profiles from single EVs (Fig. 1). Samples were collected from 30 participants with breast tumors (24 malignant, 6 benign) and 4 control participants (Fig. 1a). Single-EVs were segmented, quantified, and visualized in isolated EV samples and in situ EVs in ex vivo tissue, and a variety of metrics were extracted from these images using two-photon FLIM of NAD(P)H and SLAM microscopy. One example EV characterization dataset from a single participant with malignant breast cancer is presented in Fig. 1b–f, with the extracted optical metabolic metrics from SLAM and FLIM (Fig. 1c–f) and EV size and concentration from NTA (Fig. 1b). For optical metabolic metrics in Fig. 1c–f, each point represents a single segmented EV. The example images in Fig. 2 represent just a portion of the imaging data collected for a single participant. Single EVs can be visualized as dots (annotated with arrows) in Fig. 2, including in situ EVs in ex vivo tumor tissue (Fig. 2a,d), and isolated EVs with SLAM (Fig. 2b,e) and FLIM (Fig. 2c,f). EVs within samples from a single participant show optical metabolic heterogeneity, indicated by the broad profiles (Figs. 1d–f, 2).

This study presents the first comparison of optical metabolic signatures of single EVs from different human biofluid and tissue sources and examines their potential for use in breast cancer screening and diagnostics. EVs can be imaged in situ in excised breast biopsy tissues (Fig. 2a) and in isolated EV samples from urine and serum (Fig. 2b,c). Single EVs are segmented and quantified via a variety of nonlinear optical metrics that report on metabolic function. Two commonly used and previously validated metrics are ORR and NAD(P)H fluorescence lifetime: NAD(P)H and FAD autofluorescence intensities are used to compute the ORR (Eq. 2) and NAD(P)H fluorescence lifetime is determined (Eq. 1) for each segmented EV. In cells, ORR increases with more electron transport chain activity (FAD produced and/or NADH depleted) and decreases with increased glycolysis (NADH produced). NAD(P)H fluorescence lifetime increases when NAD(P)H is protein-bound, though the lifetime can depend on the protein^36,45^. Thus, optical metabolic metrics provide information on the metabolic state of EVs and can reflect the metabolism of the parent cells.

**Figure 3:**
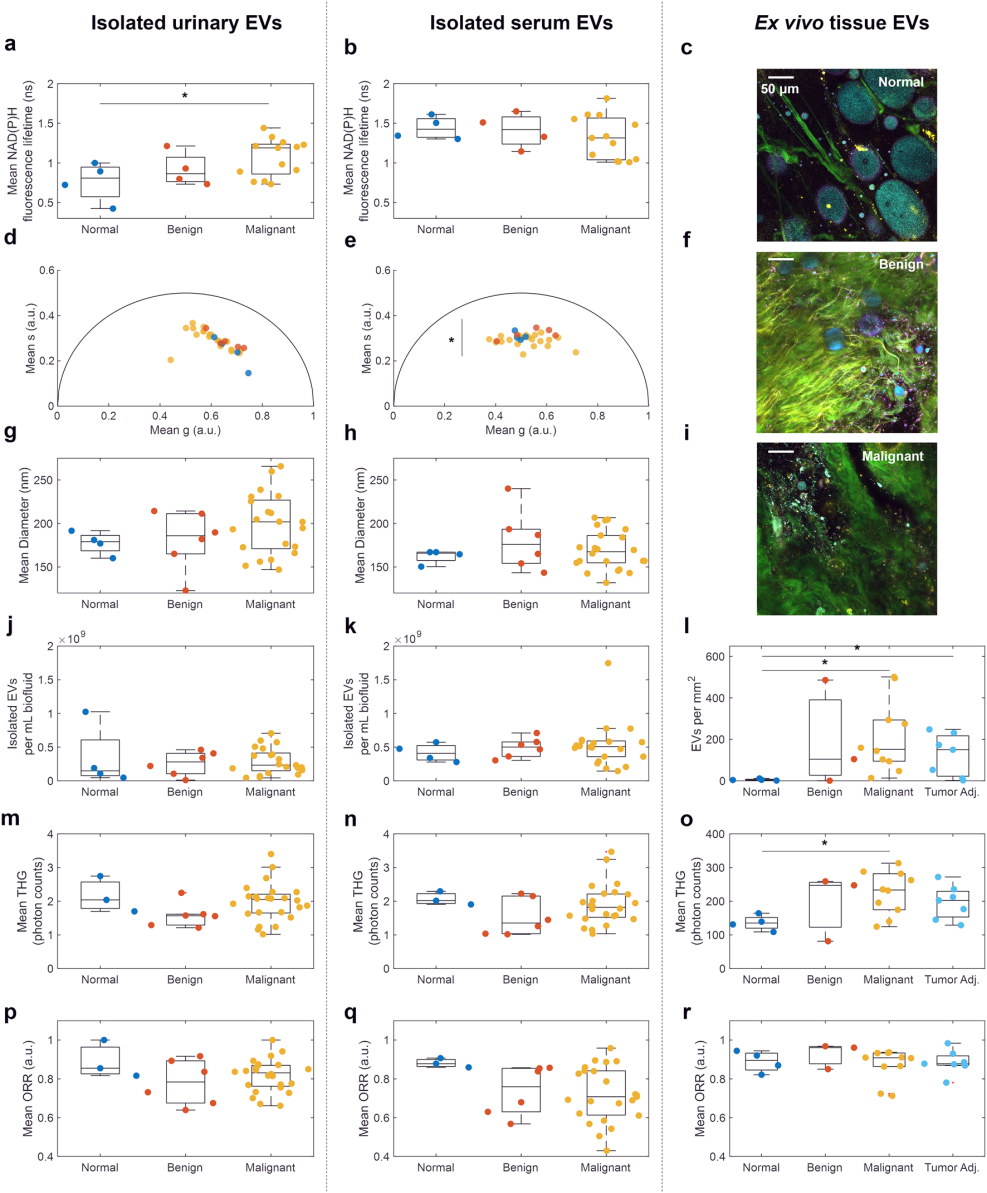
Optical metabolic characterization of control, benign, and malignant EVs across different sources. EV source is indicated by column, and patient cancer status is indicated as: normal control (blue), benign (orange), malignant (yellow), and non-tumor tissue from participants with malignant cancer “tumor adjacent” (light blue). Example SLAM images from (**c**) normal control, (**f**) benign, and (**i**) malignant ex vivo tissues are additionally shown using the same color scale. Significance: *p < 0.05; not significant if unmarked; in (**e**) the s value for benign and malignant is significantly different.

**Figure 4:**
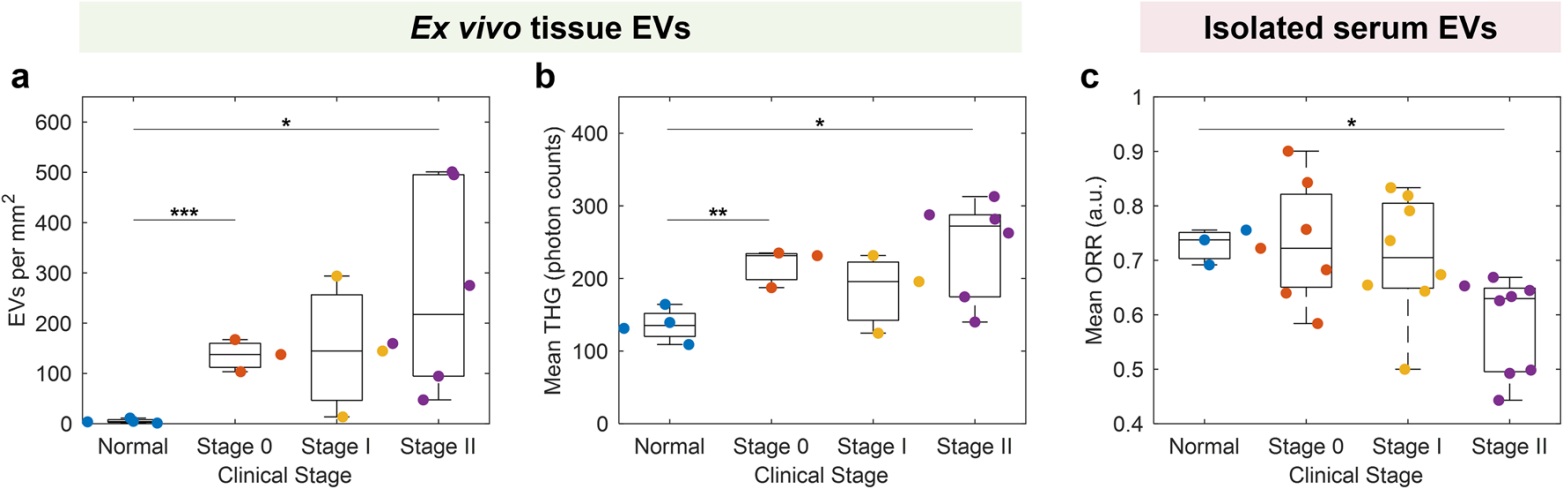
Optical metabolic characterization of EVs by cancer stage for selected metrics. Three metrics showed significant differences based on participant cancer stage: (**a**) EVs per mm^2^ segmented from ex vivo tissue images, (**b**) mean EV THG in ex vivo tissue images, and (**c**) mean EV ORR in EVs isolated from serum. Significance: *p < 0.05, **p < 0.01, ***p < 0.001, not significant if unmarked.

Within isolated EVs, seven metrics were examined for control (normal), benign, and malignant breast cancer: mean NAD(P)H fluorescence lifetime (from FLIM, Fig. 3a,b), phasor components g and s (from FLIM, Fig. 3d,e), mean diameter (from NTA, Fig. 3g,h), concentration (from NTA, Fig. 3j,k), mean THG (from SLAM, Fig. 3m,n), and mean ORR (from SLAM, Fig. 3p,q). EV signatures were also examined by clinical stage (Fig. 4, Fig. S5), tumor grade (Fig. S6), and participant age (Fig. S7). Age did not show a significant correlation with any optical metabolic metrics (Fig. S7).

### Certain EV optical metabolic signatures are dependent on cancer status in isolated EVs

Urinary EVs from participants with malignant cancer exhibited significantly elevated NAD(P)H fluorescence lifetime compared to EVs from control participants (Fig. 3a, p = 0.040), though neither showed a significant difference compared to benign cases. This significantly elevated NAD(P)H fluorescence lifetime in urinary EV samples from participants with malignant cancer compared to non-cancer controls (Fig. 3a), indicates a higher amount of NAD(P)H-related enzymatic activity in EVs from participants with malignant breast cancer. This could either be due to a compositional change: the urinary EVs from participants with malignant cancer contained more NAD(P)H-related proteins, or due to an activity change: the NAD(P)H-related proteins are significantly more active in urinary EVs in the case of malignant cancer. Metabolic reprogramming is a hallmark of cancer, and a variety of NAD(P)H-related enzymes have been implicated in key pathways associated with this reprogramming such as isocitrate dehydrogenase, glucose-6-phosphate dehydrogenase, and lactate dehydrogenase^46,47^. Urinary EVs are primarily derived from genitourinary endothelial cells^48^. For this reason, urinary EVs have predominantly been investigated for biomarkers of prostate, bladder, and kidney cancers^49^. However urinary EVs have also shown promise as biomarkers of other cancers such as non-small cell lung cancer^50^. Further studies are needed to better understand the source of this increased NAD(P)H fluorescence lifetime signature in EVs and determine if the EVs with high NAD(P)H fluorescence lifetime originate from the tumor, from local or distal immune cells, or from the kidney or bladder. Additional analysis on single-EV clustering of phasor components was performed to examine different subpopulations of EVs based on fluorescence lifetime, but did not improve performance for distinguishing cancer status (Fig. S4). No significant differences were found in the fluorescence lifetime of serum-derived EVs based on cancer presence, grade, or status.

Various studies have provided strong evidence for EVs and related particles as cancer biomarkers (including breast cancer) in blood plasma^51–53^ and serum^54^. In this study, the mean ORR in serum-derived isolated EVs from participants with stage II breast cancer, but not stage I or 0, was significantly decreased compared to control (p = 0.025, Fig. 4c). However no significant differences were found between optical metabolic metrics for EVs isolated from the serum of participants when all stages of malignant breast cancer were grouped together into the overarching category of “malignant” (Fig. 3). This may suggest that breast cancer-related optical metabolic signatures of EVs in breast cancer may develop over time and increase with disease severity since a significant difference was only visible in stage II and not the earlier stages. Accordingly, tumor-related EVs may not reach circulation until cancer has reached stage II or later stages. Lower ORR and higher NAD(P)H intensity have previously been reported in optical metabolic imaging of breast cancer cell lines, in vivo rat mammary tumors, ex vivo human breast cancer^24^, and urinary EVs in bladder cancer^29^. This shift has also been extensively reported in cells and tissues, due to the shift towards glycolytic metabolism often associated with cancer^31,33,34^. However, a lower ORR in ex vivo tissue EVs was not observed in this study, conflicting with previous results that found a higher proportion of NAD(P)H-rich (low ORR) EVs in in vivo rat mammary tumors compared to control animals^24^. This may be due to the lower tumor grades in this study or due to time-dependent changes in the optical metabolic characteristics of tissues and after removal from the body. Previous studies showed that optical metabolic signatures of isolated EVs can change after 48 h of storage at − 20 °C^29^ and that in vivo vs. ex vivo imaging can alter optical metabolic metrics of EVs in intact tissue^28^. Additionally, previous work on breast cancer found more significant changes in ORR and NAD(P)H in the later stages (II and III) of cancer^24^, whereas this study primarily recruited earlier stage participants and did not have any participants with stage III breast cancer (Table S1, Fig. S1). Despite the trends of lower ORR in participants with malignant cancer compared to control participants in urinary EVs, no statistical significance was found in urinary EV ORR between normal, malignant, and benign cases (Fig. 3p), between clinical stages (Fig. S5a), or between tumor grade (Fig. S6a), which in part could be due to the small number of samples or the relatively larger contribution of non-tumor related EVs in urinary samples.

### EV concentration and THG signature are dependent on cancer status for in situ EVs

In ex vivo tissue samples, three EV metrics were examined from SLAM images: concentration in EVs per mm^2^ (Fig. 3l), mean THG (Fig. 3o), and mean ORR (Fig. 3r). The segmented EV density was significantly higher in malignant tumor tissue (p = 0.039) and adjacent non-tumor tissue (p = 0.049) from participants with malignant cancer compared to tissue from control participants (Fig. 3l). Furthermore, the EV THG intensity is higher in the malignant tumor images compared to the control images (Fig. 3o, p = 0.022). Similar to Fig. 3l,o, EVs per mm^2^ and EV THG signature were both significantly elevated in stage 0 and stage II cancer compared to controls (Fig. 4a,b). When grouped by tumor grade, similar trends emerged in ex vivo tissue EVs per mm^2^ and THG (Fig. S6c). This increased EV concentration in tumors has previously been observed using label-free nonlinear optical microscopy in ex vivo breast cancer biopsy specimens^27^. It also corresponds well with studies which found that cancer cells produce more EVs, especially in the case of hypoxia^55^. THG signal increases with particle diameter in index-matched and non-index-matched solvents^56^. Thus, this increase in THG signature in tumor tissue EVs could be due to the increased size of cancer-related EVs, sometimes termed large oncosomes^57^. However, no strong positive correlation was found between the size of isolated EVs and their THG signature, which suggest that the size of EVs isolated from urine and serum was not increased (Fig. 3), indicating that this increased THG signature is more relevant in tissue, and not associated with the size of isolated EVs.

### All optical metabolic signatures are dependent on EV source

**Figure 5:**
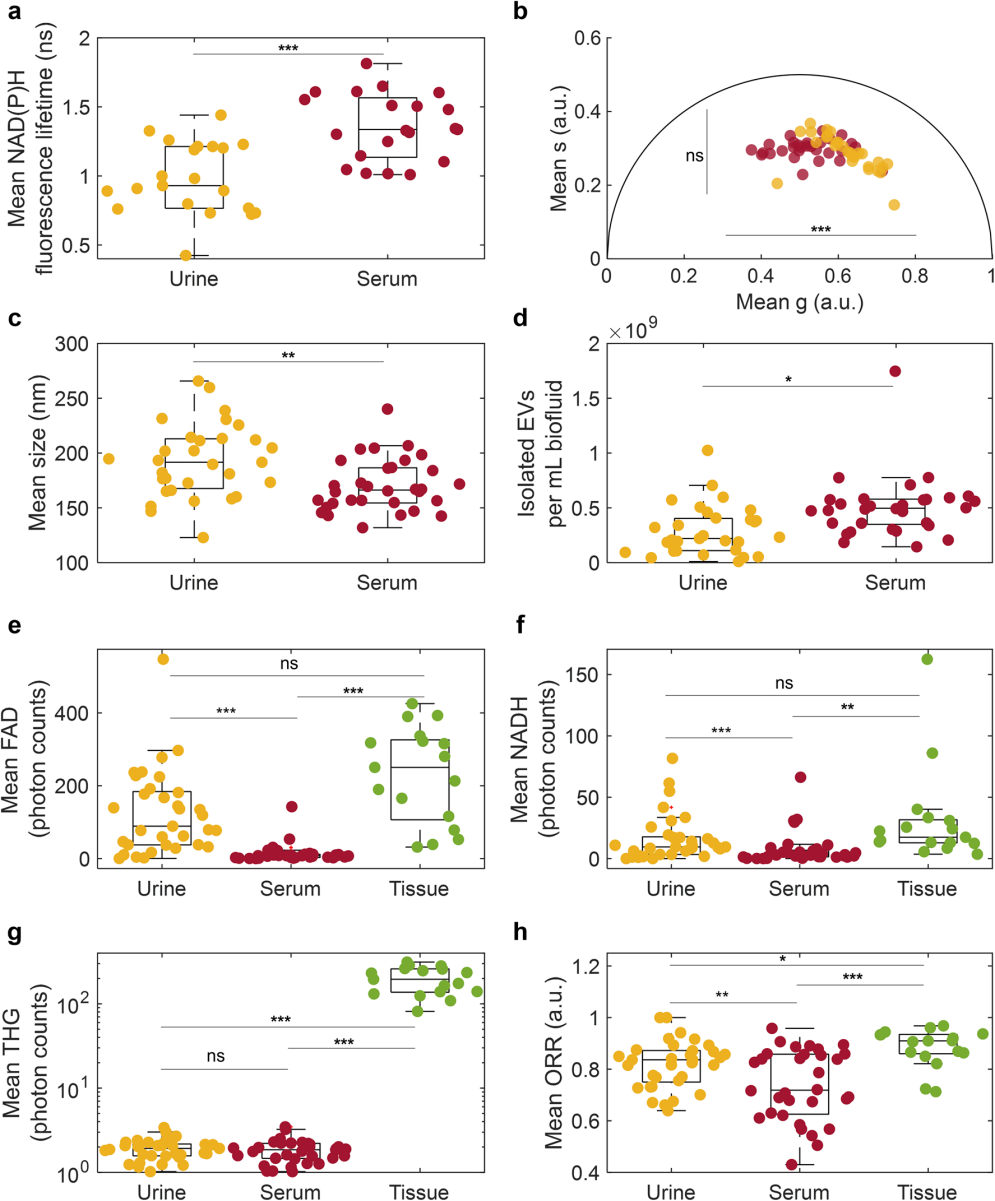
Optical metabolic characterization of EVs from different sources. The mean value of each metric was computed for measured EVs from all sources across all cancer status groups for: (**a**) mean NAD(P)H fluorescence lifetime from FLIM, (**b**) g and s phasor lifetime components from FLIM, (**c**) mean size from NTA, (**d**) concentration of isolated EVs per mL of initial biofluid from NTA, (**e**) mean FAD SLAM intensity, (**f**) mean NAD(P)H SLAM intensity, (**g**) mean THG SLAM intensity, and (**h**) mean ORR, calculated from NAD(P)H and FAD intensities from SLAM. Significance using paired Student’s t-test to examine intra-individual changes: *ns* not significant; *p < 0.05, **p < 0.01, ***p < 0.001.

In order to assess how optical metabolic signatures depend on EV source (urine, serum, tissue), paired Student’s t-tests were used to compare the signatures across all metrics for all participants. NAD(P)H fluorescence lifetime and phasor component g showed significant differences between serum and urine, with significantly higher fluorescence lifetimes in serum samples, indicating a larger presence of protein-bound NAD(P)H (Fig. 5a,b, p < 0.001), which has a longer lifetime than free NAD(P)H^36–38^. Furthermore, compared to serum-derived EVs, isolated urinary EVs were significantly larger in mean diameter (Fig. 5c, p = 0.004) and had lower concentration per mL of original biofluid (Fig. 5d, p = 0.020), as determined by NTA. FAD and NAD(P)H intensities were significantly lower in serum-derived isolated EVs compared to urinary isolated EVs and ex vivo tissue EVs measured in situ (Fig. 5e,f, p < 0.001). THG signature was significantly elevated in ex vivo tissue EVs in situ, averaging 105 times higher intensity than the isolated EVs (Fig. 5g, p < 0.001). The ORR was significantly different between all sources, with the lowest ORR in serum-derived EVs and the highest ORR in tissue EVs (Fig. 5h). Thus, clear differences between EVs isolated from urine and serum emerged from almost all variables, as well as between the two biofluid-based isolated EVs and ex vivo tissue EVs (Fig. 5). The origin of the EV populations in biofluids and tissue varies greatly, which likely accounts for the dramatic optical differences.

**Figure 6:**
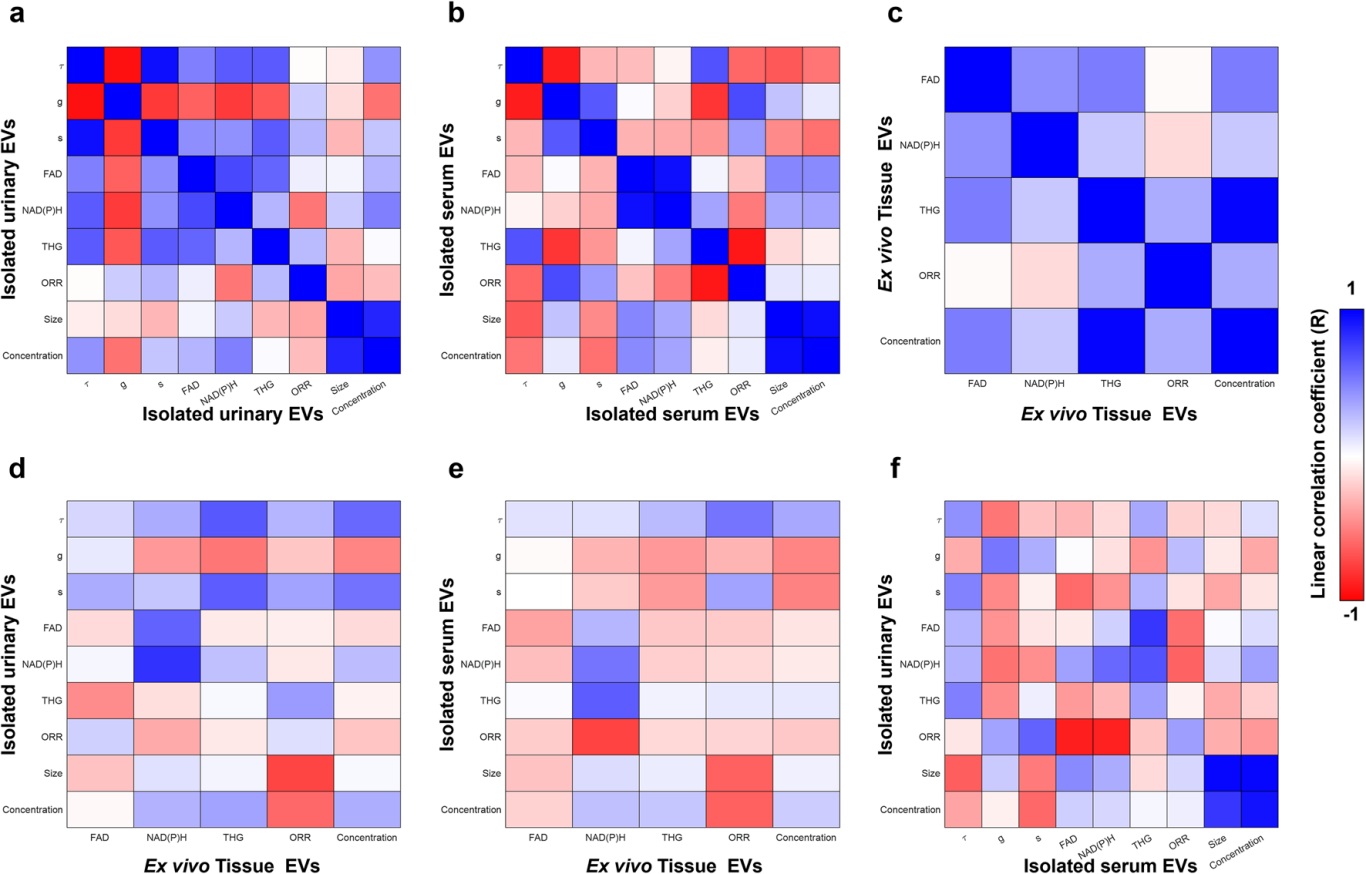
Linear correlation coefficient examining optical metabolic variables within and between EV source. Correlations within a single-source for: (**a**) isolated urinary EVs, (**b**) isolated serum EVs, and (**c**) ex vivo tissue EVs. Correlations comparing different sources for: (**d**) isolated urinary EVs vs. ex vivo tissue EVs, (**e**) isolated serum EVs vs. ex vivo tissue EVs, and (**f**) isolated urinary EVs vs. isolated serum EVs.

Despite these differences, a variety of variables showed high linear correlation across different EV sources from the same participant (Fig. 6, Table 1). The linear correlation coefficient was calculated for all metrics within (Fig. 6a–c) and between (Fig. 6d–f) EV sources. When examining the correlation coefficients, it is important to note that ORR is calculated from FAD and NAD(P)H intensities (Eq. 2) and mean lifetime (τ) is calculated from g and s values (Eq. 1), causing stronger linear relationships between these variables since they are dependent. Selected high absolute value (> 0.7, < − 0.7) correlation coefficients are listed in Table 1. All coefficients are listed in Tables S4–S9. Notably, NAD(P)H intensity is highly positively correlated between urinary EVs and tissue EVs (R = 0.798), pointing to a link between tumor EVs and urinary EVs. Serum EV ORR is highly negatively correlated with tissue EV NAD(P)H intensity (R = -0.737); since ORR decreases with increased NAD(P)H intensity, this further strengthens the link between NAD(P)H signatures across EV sources. Similarly, urinary EV ORR is highly negatively correlated with serum EV NAD(P)H (R = − 0.874). Overall, these results suggest that some optical metabolic metrics may be conserved across different EV sources, which warrants future investigation.

**Table 1:** Highly correlated optical metabolic markers within and between EV sources.

Optical metabolic metric 1	Optical metabolic metric 2	Linear correlation coefficient R
Urine NAD(P)H intensity	Urine g	− 0.771
Urine NAD(P)H intensity	Urine FAD intensity	0.712
Serum THG intensity	Serum g	− 0.791
Serum ORR intensity	Serum g	0.705
Serum ORR intensity	Serum THG intensity	− 0.912
Tissue THG intensity^+^	Tissue EV concentration^+^	0.982
Urine NAD(P)H intensity	Tissue NAD(P)H intensity	0.798
Serum ORR	Tissue NAD(P)H intensity	− 0.737
Urine ORR	Serum FAD intensity	− 0.880
Urine ORR	Serum NAD(P)H intensity	− 0.874
Urine FAD intensity	Serum THG intensity	0.785

Optical metabolic metrics with high (R > 0.7 or R < − 0.7) linear correlation coefficients. Size and concentration determined with NTA and relationships between variables directly dependent based on Eqs. 1 and 2 were not included.

^+^THG channel is used for EV segmentation, so tissue THG intensity and EV concentration are not independent.

### Implications for future work on label-free nonlinear optical microscopy of EVs

This preliminary study had some limitations. Primarily, the study population was small, which means that the results may not be universal, and that these small numbers impact the statistical significance of the findings. The significantly elevated NAD(P)H fluorescence lifetime in urinary EVs (Fig. 3a) and lower ORR in serum-derived EVs (Fig. 4c), support the potential for optical metabolic characterization of EVs in liquid biopsy and should be confirmed on a larger scale. Additionally, this study focused only on early-stage breast cancer. While this population is important for screening and diagnostics, only examining early-stage participants means that the results for later stages of breast cancer could show a different outcome. Also, various factors in urine and blood collection may impact results, such as the time of the day, medications or supplements, or the presence of other diseases or conditions.

A limitation for the translation of nonlinear optical microscopy for clinical use is the high cost of equipment and specialized skills needed to build and operate these systems. Future work could examine the possibility to characterize single-photon NAD(P)H and FAD autofluorescence with lower cost laboratory equipment such as confocal and widefield micro-scopes, which are more accessible and can be used for EV analysis, as has been demonstrated with label-based fluorescence techniques for EV imaging^58^. However, the longer wavelengths used for excitation in nonlinear optics enable better depth penetration into biological tissue, which would be lost when using a single-photon fluorescence imaging system. Also, future work could incorporate NAD(P)H and/or FAD FLIM into a SLAM channel to enable colocalization of all metrics.

Another aspect for future investigation is the relative abundance of cancer-related EVs in biofluids. Future work could involve the use of tumor-specific labels or markers to examine what subpopulations of EVs in urine and serum are from the tumor, and investigate other suspected origins such as blood, immune, and bladder cells. Nonlinear optical microscopy can also provide spatial and temporal information in vivo and could be used to examine EV trafficking throughout the body and in and around the tumor microenvironment. By observing the distribution, transfer, and transport of EVs between tissues and the blood and lymphatic circulatory systems, a better understanding of the relationship between EVs in tissues and different biofluids could be achieved.

## Conclusions

Nonlinear optical microscopy is a novel tool with great potential to examine EVs from a variety of different sources, including isolated EVs and in situ EVs in ex vivo tissue samples. It enables direct comparison of the optical metabolic properties from multiple EV sources to evaluate their potential as biomarkers and the relationship between EVs from these different sources. Overall, isolated urinary EV autofluorescence lifetime of NAD(P)H and ex vivo tissue EV density were significantly elevated in cases of malignant tumors, while the ORR of isolated serum-derived EVs showed significant changes in stage II of malignant cancer, but not in earlier stages of malignant cancer. EV optical metabolic properties were highly dependent on whether the EVs were in intact breast tissue or were isolated from serum or urine. Despite that, EV optical signatures showed some correlations in NAD(P)H autofluorescence and ORR values that could indicate some properties of individual EVs are conserved despite differences in source. Further investigations are needed to better understand the relationship between EV source and optical metabolic properties.

### Supplementary Information

Supplementary Information.

## Data Availability

The data and analysis codes generated in this study will be made publicly available upon publication via Open Science Framework at: https://osf.io/d37ac/?view_only=523ce62c46aa4f3b8524c1b13a2c4255^59^.
