# Supplementary material for: Label-free nonlinear optical signatures of extracellular vesicles in liquid and tissue biopsies of human breast cancer
Source: Sci Rep. 2024 Mar 6;14:5528. doi: 10.1038/s41598-024-55781-4 (PMC10917806; doi:10.1038/s41598-024-55781-4)
Supplement: Supplementary file 1 — Supplementary Information. [file 41598_2024_55781_MOESM1_ESM.docx]

*Supplementary Material for:*

Label-free nonlinear optical signatures of extracellular vesicles in liquid and tissue biopsies of human breast cancer

Janet E. Sorrells^1,2, ⸸^, Jaena Park^1,2, ⸸^, Edita Aksamitiene^1, ⸸^, Marina Marjanovic^1,2,3^, Elisabeth M. Martin^2^, Eric J. Chaney^1,4^, Anna M. Higham^5^, Kimberly A. Cradock^5^, Zheng G. Liu^5^, Stephen A. Boppart^1,2,3,4,6,7,*^

^1^Beckman Institute for Advanced Science and Technology, University of Illinois Urbana-Champaign, Urbana, IL, 61801, USA

^2^Department of Bioengineering, University of Illinois Urbana-Champaign, Urbana, IL, 61801, USA

^3^NIH/NIBIB P41 Center for Label-free imaging and Multiscale Biophotonics (CLIMB), University of Illinois Urbana-Champaign, Urbana, IL, 61801, USA

^4^Cancer Center at Illinois, Urbana, IL, 61801, USA

^5^Carle Foundation Hospital, Urbana, IL, 61801, USA

^6^Department of Electrical and Computer Engineering, University of Illinois Urbana-Champaign, Urbana, IL, 61801, USA

^7^Interdisciplinary Health Sciences Institute, University of Illinois Urbana-Champaign, Urbana, IL, 61801, USA

^⸸^These authors contributed equally to this work.

*Corresponding author: boppart@illinois.edu

**Table S1.** Subject demographics and relevant health information. Abbreviations: DCIS, ductal carcinoma *in situ*; LCIS, lobular carcinoma *in situ*; IDC, invasive ductal carcinoma; ILC, invasive lobular carcinoma; uEVs, urinary EVs; sEVs, serum EVs.

| **#** | **Gender** | **Age** | **Type of cancer** | **Cancer status** | **Clinical staging of cancer** | **Tumor differentiation grade** | **Previous malignancy** | **Current kidney problems** | **History of urinary or kidney problems** | **Biofluid EVs imaged** | | | | **Tissues imaged** |  |
| --- | --- | --- | --- | --- | --- | --- | --- | --- | --- | --- | --- | --- | --- | --- | --- |
| **1** | **F** | 52 | DCIS, LCIS | Malignant | I | 2 | No | N/A | N/A | SLAM: uEVs, sEVs | | | |  |  |
| **2** | **F** | 82 | IDC, DCIS | Malignant | IIA | 3 | Yes | No | Yes (chronic kidney disease) | SLAM: uEVs, sEVs | | | |  |  |
| **3** | **F** | 81 | IDC, DCIS | Malignant | IIA | 2 | No | No | No | SLAM: uEVs, sEVs; FLIM: uEVs, sEVs | | | | SLAM: tumor, tumor adjacent |  |
| **4** | **F** | 89 | IDC, ILC, DCIS | Malignant | IIB | 3 | No | No | Yes (chronic kidney disease) | SLAM: uEVs, sEVs; FLIM: uEVs, sEVs | | | | SLAM: tumor, tumor adjacent |  |
| **5** | **F** | 73 | IDC, DCIS | Malignant | IIA | 1 | No | No | No | SLAM: uEVs, sEVs; FLIM: uEVs, sEVs | | | | SLAM: tumor |  |
| **6** | **F** | 71 | IDC, DCIS | Malignant | IIA | 3 | No | No | Yes (kidney stones) | SLAM: uEVs, sEVs; FLIM: uEVs, sEVs | | | | SLAM: tumor, tumor adjacent |  |
| **7** | **F** | 53 | IDC, DCIS | Malignant | IIA | 3 | No | Yes (UTI on ciprofloxacin, urine is clear) | Yes (kidney stones) | SLAM: uEVs, sEVs; FLIM: uEVs, sEVs | | | | SLAM: tumor |  |
| **8** | **F** | 38 | IDC, DCIS | Malignant | I | 3 | No | N/A | N/A | SLAM: uEVs, sEVs; FLIM: sEVs | | | | SLAM: tumor adjacent |  |
| **9** | **F** | 34 |  | Benign |  | 0 | No | No | No | SLAM: uEVs, sEVs; FLIM: uEVs, sEVs | | | |  |  |
| **10** | **F** | 70 | IDC, ILC, DCIS | Malignant | IIA | 1 | Yes (resection) | No | No | SLAM: uEVs, sEVs; FLIM: uEVs, sEVs | | | |  |  |
| **11** | **F** | N/A | DCIS, LCIS | Malignant | 0 | 2 | Yes (other breast) | N/A | N/A | SLAM: uEVs, sEVs | | | |  |  |
| **12** | **F** | 64 | IDC, DCIS | Malignant | I | 2 | Yes (lung) | No | Yes (kidney stones) | SLAM: uEVs, sEVs | | | |  |  |
| 13 | M | 71 | IDC, DCIS | Malignant | I | 2 | Yes (breast) | No | Yes (glomerular nephritis at age 8) | SLAM: uEVs, sEVs; FLIM: uEVs, sEVs | | | | SLAM: tumor adjacent |  |
| **14** | **F** | 43 |  | Benign |  | 0 | No | No | No | SLAM: uEVs, sEVs; FLIM: uEVs, sEVs | | | | SLAM: tumor (benign) |  |
| **15** | **F** | 60 | IDC, DCIS | Malignant | I | 3 | No | No | No | SLAM: uEVs, sEVs | | | |  |  |
| **16** | **F** | 57 | IDC, DCIS | Malignant | I | 1 | No | No | No | SLAM: uEVs, sEVs; FLIM: uEVs, sEVs | | | | SLAM: tumor, tumor adjacent |  |
| **17** | **F** | 52 |  | Benign |  | 0 | No | No | No | SLAM: uEVs, sEVs; FLIM: uEVs, sEVs | | | |  |  |
| **18** | **F** | 64 | IDC | Malignant | IB | 3 | No | No | Yes (protein in urine) | SLAM: uEVs, sEVs; FLIM: uEVs, sEVs | | | | SLAM: tumor, tumor adjacent |  |
|  |  |  |  |  |  |  |  |  |  |  |  |  |  |  |  |
| **19** | **F** | 52 | IDC, DCIS | Malignant | I | 2 | No | No | Yes (kidney infection 20 years ago) | SLAM: uEVs, sEVs; FLIM: uEVs, sEVs | | | | SLAM: tumor, tumor adjacent |  |
| **20** | **F** | 56 | LCIS | Benign | 0 | 0 | Yes (uterus) | No | Yes | SLAM: uEVs, sEVs | | | | SLAM: tumor (benign) |  |
|  |  |  |  |  |  |  |  |  |  |  |  |  |  |  |  |
| **21** | **F** | 51 | DCIS | Benign | 0 | 3 | No | No | No | SLAM: uEVs, sEVs; FLIM: uEVs, sEVs | | | |  |  |
|  |  |  |  |  |  |  |  |  |  |  |  |  |  |  |  |
| **22** | **F** | 73 | DCIS | Malignant | 0 | 0 | Yes (breast) | No | Yes | SLAM: uEVs, sEVs | | | |  |  |
| **23** | **F** | 62 | lobular hyperplasia | Benign | 0 | 0 | No | No | No | SLAM: uEVs, sEVs; FLIM: uEVs, sEVs | | | | SLAM: tumor (benign) |  |
|  |  |  |  |  |  |  |  |  |  |  |  |  |  |  |  |
| **24** | **F** | 62 | DCIS | Malignant | 0 | 0 | Yes (other breast) | No | No | SLAM: uEVs, sEVs; FLIM: uEVs, sEVs | | | | SLAM: tumor, tumor adjacent |  |
| **25** | **F** | 18 | __ | Normal | __ | __ | No | No | No | SLAM: uEVs, sEVs; FLIM: uEVs, sEVs | | | | SLAM: tissue (normal) |  |
|  |  |  |  |  |  |  |  |  |  |  |  |  |  |  |  |
| **26** | **F** | 22 | __ | Normal | __ | __ | No | No | No | SLAM: uEVs, sEVs; FLIM: uEVs, sEVs | | | | SLAM: tissue (normal) |  |
|  |  |  |  |  |  |  |  |  |  |  |  |  |  |  |  |
| **27** | **F** | 50 | __ | Normal (some ductal hyperplasia) | __ | __ | Yes (cervical cancer 25 years ago) | No | Yes (kidney stones) | SLAM: uEVs, sEVs; FLIM: uEVs, sEVs | | | | SLAM: tissue (normal) |  |
|  |  |  |  |  |  |  |  |  |  |  |  |  |  |  |  |
| **28** | **F** | 78 | DCIS | Malignant | 0 | 0 | Yes (lung) | No | No | SLAM: uEVs, sEVs; FLIM: uEVs, sEVs | | | |  |  |
| **29** | **F** | 72 | DCIS | Malignant | 0 | 0 | Yes | No | Yes (kideny stones, cyst tumor on kidney) | SLAM: uEVs, sEVs; FLIM: uEVs, sEVs | | | |  |  |
| **30** | **F** | 42 | __ | Normal | __ | __ | No | No | No | FLIM: uEVs, sEVs | | | | SLAM: tissue (normal) |  |
|  |  |  |  |  |  |  |  |  |  |  |  |  |  |  |  |
| **31** | **F** | 51 | DCIS | Malignant | I | 2 | Yes (DCIS in the other breast) | No | No | SLAM: uEVs, sEVs; FLIM: uEVs, sEVs | | | | SLAM: tumor |  |
|  |  |  |  |  |  |  |  |  |  |  |  |  |  |  |  |
| **32** | **F** | 67 | DCIS | Malignant | I | 1 | Yes (breast cancer) | No | No | SLAM: uEVs, sEVs; FLIM: uEVs, sEVs | | | |  |  |
| **33** | **F** | 63 | IDC, DCIS | Malignant | I | 3 | Yes (basal cell carcinoma) | No | No | SLAM: uEVs, sEVs; FLIM: uEVs, sEVs | | | |  |  |
| **34** | **F** | 57 | IDC, DCIS | Malignant | II | 2 | No | No | No | SLAM: uEVs, sEVs; FLIM: uEVs, sEVs | | | | SLAM: tumor, tumor adjacent |  |
|  |  |  |  |  |  |  |  |  |  |  |  |  |  |  |  |


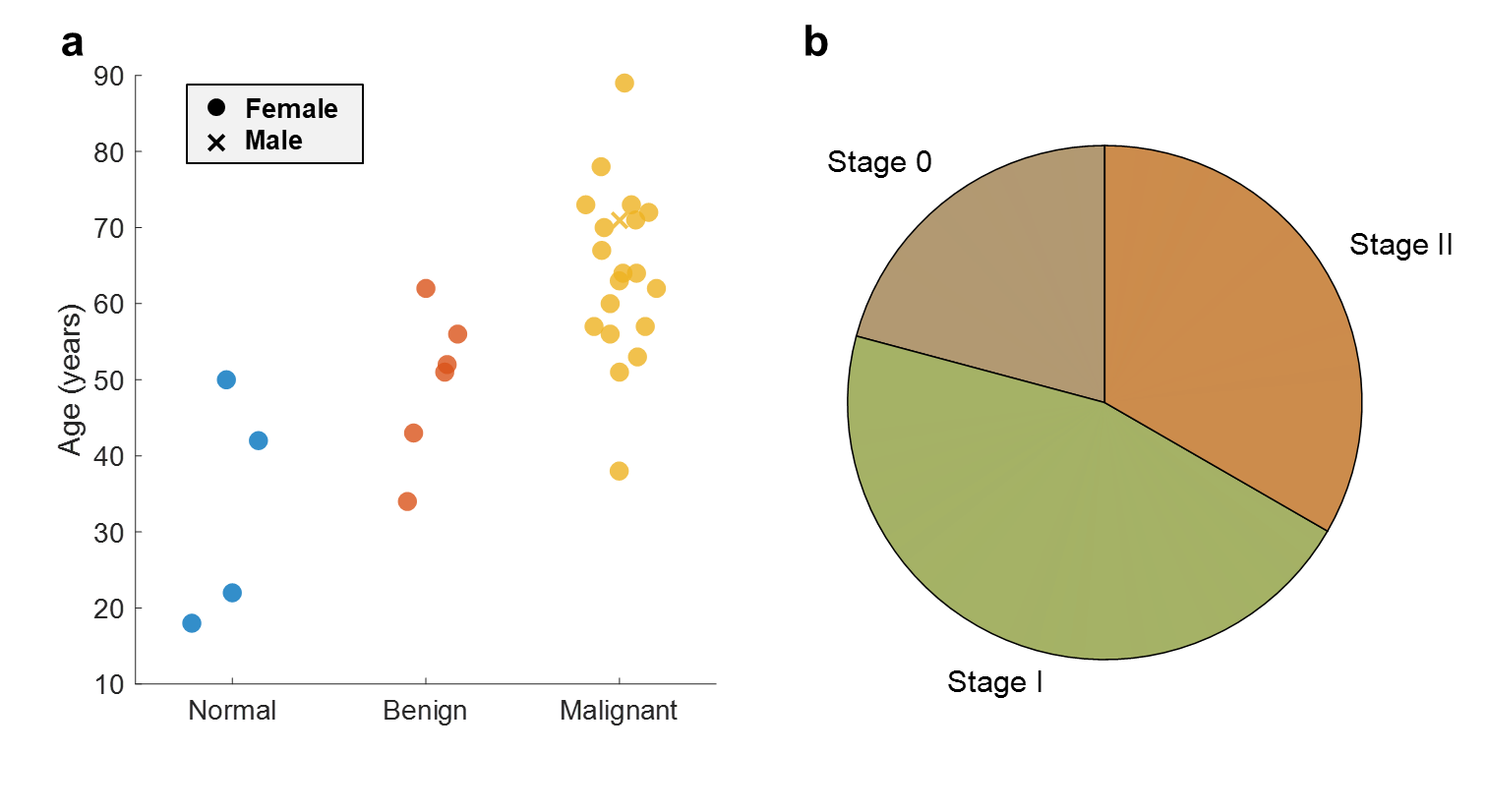


**Figure S1.** Visual representation of participant demographics and cancer stage. (a) Subject age, cancer status, and gender. (b) Cancer stage for subjects with malignant cancer.

**Table S2.** Volume of biofluid collected for each participant.

| **Participant** **#** | **Urine Volume (mL)** | **Serum Volume (mL)** |
| --- | --- | --- |
| 1 | 31 | 2 |
| 2 | 45 | 3 |
| 3 | 37.5 | 5 |
| 4 | 75 | 5 |
| 5 | 44 | 4.5 |
| 6 | 80 | 4 |
| 7 | 50 | 3.5 |
| 8 | 80 | 4.5 |
| 9 | 40 | 4 |
| 10 | 40 | 3.4 |
| 11 | 95 | 3 |
| 12 | 35 | 4.55 |
| 13 | 77 | 3.5 |
| 14 | 37.5 | 2.5 |
| 15 | 20 | 4 |
| 16 | 32.5 | 4.5 |
| 17 | 2.5 | 4.5 |
| 18 | 23 | 3.5 |
| 19 | 35 | 3 |
| 20 | 38 | 4.435 |
| 21 | 42.5 | 3 |
| 22 | 42 | 3.6 |
| 23 | 36 | 3.7 |
| 24 | 25 | 4 |
| 25 | 84 | 4 |
| 26 | 30 | 5.2 |
| 27 | 30 | 4 |
| 28 | 50 | 2.9 |
| 29 | 23.5 | 3.5 |
| 30 | 45 | 4.7 |
| 31 | 82 | 4 |
| 32 | 64 | 4.15 |
| 33 | 26 | 3.25 |
| 34 | 25 | 1.8 |
| **Mean** | **44.8** | **3.77** |
| **Standard Deviation** | **22.0** | **0.82** |

**
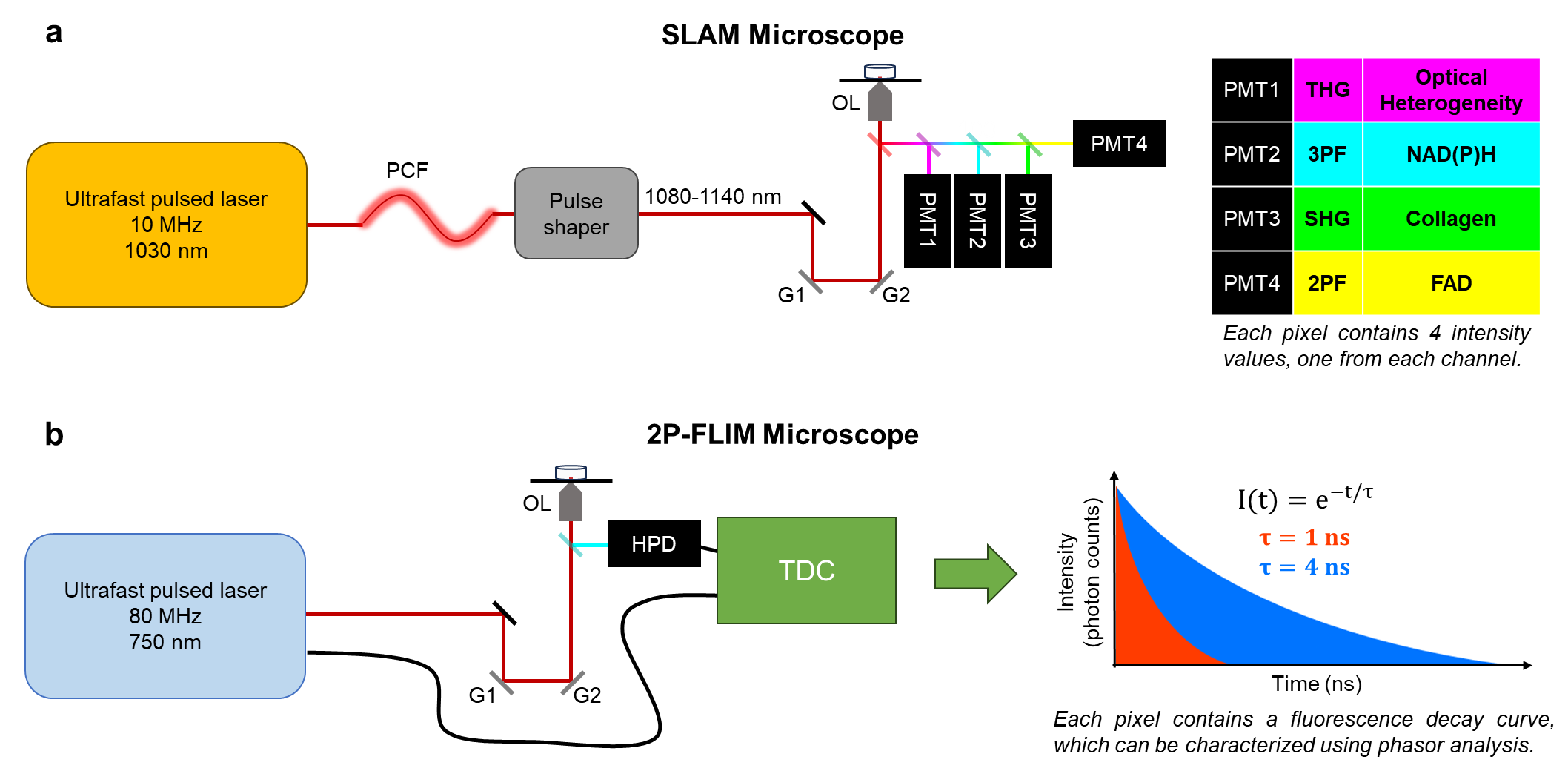
**

**Figure S2.** Condensed system diagrams for custom microscopy systems used for EV imaging. (a) SLAM microscopy setup, including the four different channels. (b) Two-photon FLIM microscopy, which uses a time-to-digital converter (TDC) to time-tag incoming photons and build a fluorescence decay curve for each pixel. SLAM: simultaneous label-free autofluorescence multiharmonic, FLIM: fluorescence lifetime imaging microscopy, PCF: photonic crystal fiber, G1/G2: galvanometer scanning mirrors, OL: objective lens, PMT: photomultiplier tube, THG: third harmonic generation, 3PF: three-photon fluorescence, SHG: second harmonic generation, 2PF: two-photon fluorescence, HPD: hybrid photodetector, TDC: time-to-digital converter.

**Table S3.** Total number of EVs imaged per sample. If 10 or fewer EVs were imaged, the data was excluded from analysis.

| **Participant** **#** | **Urinary EV FLIM** | **Urinary EV SLAM** | **Serum EV FLIM** | **Serum EV SLAM** | **Tumor *ex vivo* EVs** | **Non-tumor *ex vivo* EVs** |
| --- | --- | --- | --- | --- | --- | --- |
| 1 |  | 105 |  | 346 |  |  |
| 2 |  | 5352 |  | 2960 |  |  |
| 3 | 347 | 463 | 174 | 403 | 7092 | 11025 |
| 4 | 62 | 357 | 151 | 475 | 2107 | 1449 |
| 5 | 486 | 694 | 341 | 351 | 61140 |  |
| 6 | 278 | 479 | 74 | 1092 | 33957 | 21356 |
| 7 |  | 59 | 97 | 33 | 4203 |  |
| 8 | 174 | 1573 | 82 | 2368 |  |  |
| 9 | 484 | 389 | 39 | 495 |  |  |
| 10 | 2654 | 305 | 121 | 265 |  |  |
| 11 |  | 56 |  | 60 |  |  |
| 12 |  | 14 |  | 20 |  |  |
| 13 | 815 | 23 | 44 | 6 |  |  |
| 14 | 2502 | 3500 | 32 | 35 | 26411 |  |
| 15 | 584 | 134 | 141 | 129 |  |  |
| 16 | 25 | 109 | 77 | 8918 | 17854 | 6539 |
| 17 | 571 | 108 | 319 | 126 |  |  |
| 18 | 7340 | 429 | 128 | 131 | 36254 | 18525 |
| 19 | 4024 | 297 | 82 | 272 |  |  |
| 20 |  | 144 |  | 88 | 6119 |  |
| 21 | 85 | 119 | 142 | 215 |  |  |
| 22 | 734 | 11 | 98 | 33 |  |  |
| 23 | 4452 | 182 | 455 | 255 | 20656 |  |
| 24 | 1649 |  | 55 |  | 12771 | 246 |
| 25 | 1297 |  | 100 | 38 |  | 463 |
| 26 | 1482 | 13 | 128 | 42 |  | 589 |
| 27 | 492 |  | 72 | 40 |  | 1394 |
| 28 | 2553 |  | 66 | 15 |  |  |
| 29 | 264 |  | 17 |  |  |  |
| 30 | 42 |  | 83 |  |  | 318 |
| 31 | 2791 | 12 | 77 |  | 602 |  |
| 32 | 313 |  | 48 |  |  |  |
| 33 | 18 |  | 476 |  |  |  |
| 34 | 735 | 121 | 164 | 139 | 121232 | 28599 |
| **Mean** | **1330** | **579** | **134** | **691** | **26954** | **8228** |
| **Standard Deviation** | **1700** | **1206** | **117** | **1753** | **33134** | **10206** |

**
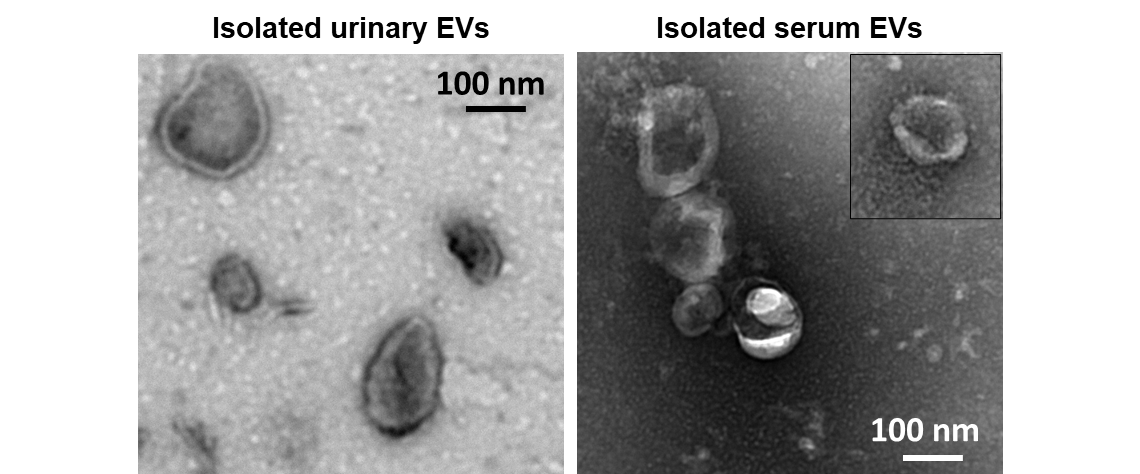
**

**Figure S3.** Validation of EV isolation via transmission electron microscopy (TEM) for urinary and serum EVs. Isolated serum EVs were sparse so selections from two fields-of-view are shown.

**
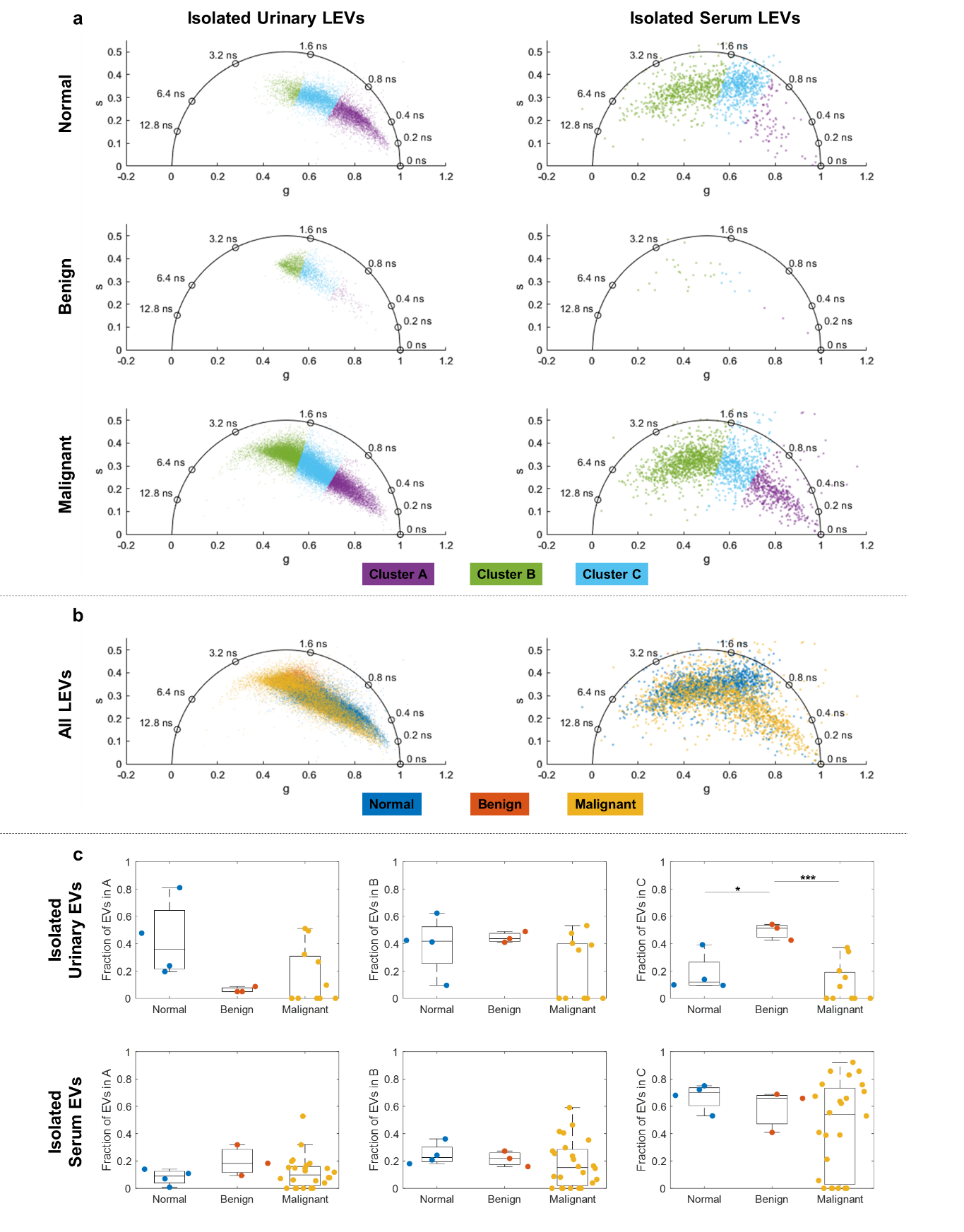
**

**Figure S4.** K-means clustering of single-EV fluorescence lifetime profiles. All phasor components for isolated EVs were pooled together and clustered using K-means clustering with k = 3. (a) The three clusters (A,B,C) are indicated with corresponding colors on the phasor plots for each different group. (b) All data for each biofluid is shown with corresponding color for subject cancer status. (c) Relative fractions (by # of EVs) of EVs in each cluster by subject cancer status. Significance: * p < 0.05, ** p < 0.01, *** p < 0.001; not significant if unmarked.

**
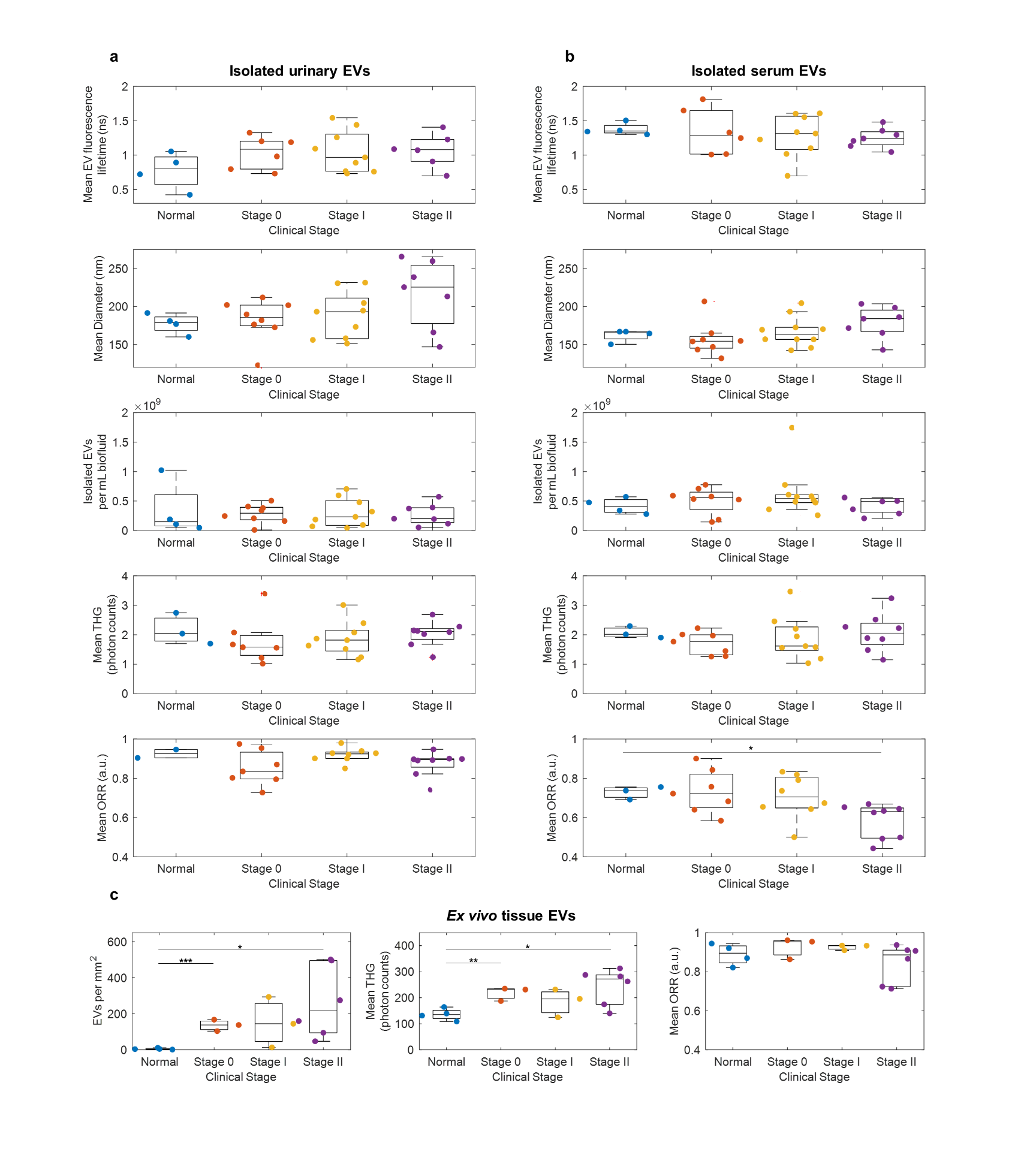
**

**Figure S5.** Relationship between EV signatures and clinical stage. EV signatures from (a) urine, (b) serum, and (c) *ex vivo* tissue separated by clinical stage of cancer. Tumor adjacent tissue was not considered.

**
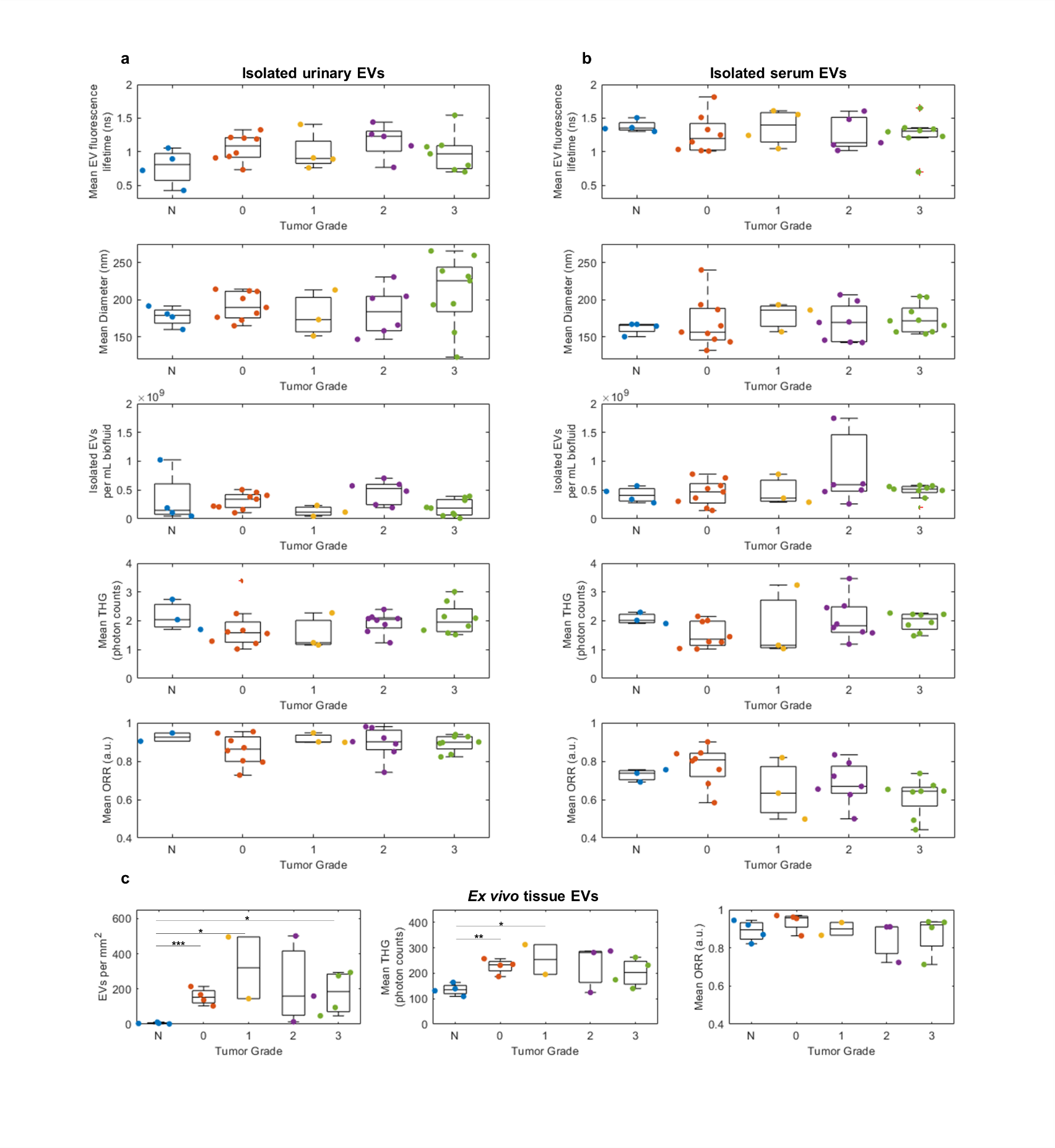
**

**Figure S6.** Relationship between EV signatures and tumor grade. EV signatures from (a) urine, (b) serum, and (c) *ex vivo* tissue separated by tumor grade. Tumor adjacent tissue was not considered. N indicates normal control (no tumor), otherwise tumor grade was determined by pathologist.

**
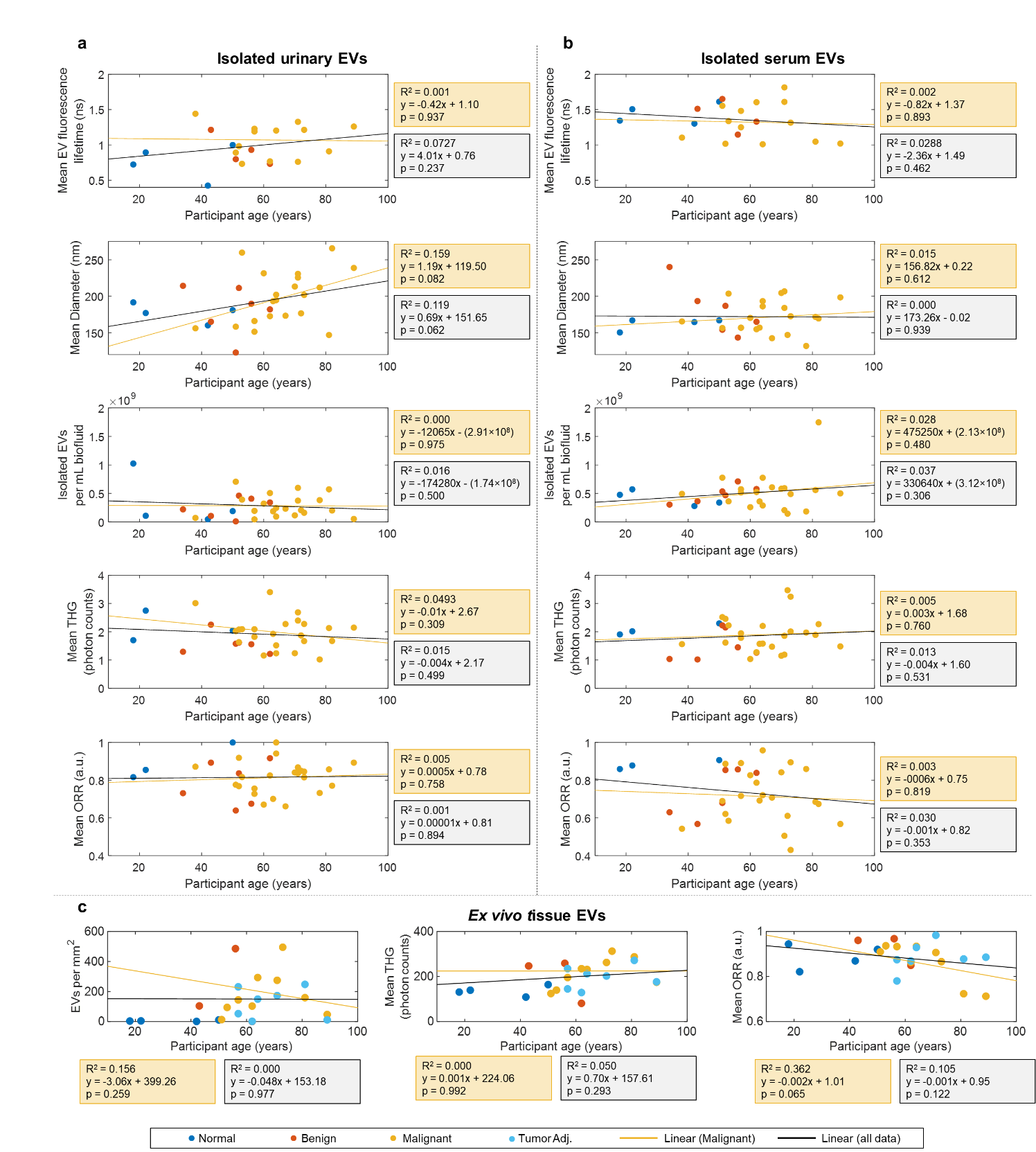
**

**Figure S7.** Relationship between EV signatures by participant age. EV signatures from (a) urine, (b) serum, and (c) *ex vivo* tissue were examined by cancer status (dark blue: normal control, orange: benign; yellow: malignant; light blue: non-tumor tissue from patient with malignant tumor) and participant age. Parameters for linear fits corresponding to only the malignant group (yellow) and all data pooled together (black/gray) are shown with corresponding plots. No significant linear relationship was found across all variables.

**Table S4.** Isolated urinary EV linear correlation coefficients.

|  | **τ** | **g** | **s** | **FAD** | **NAD(P)H** | **THG** | **ORR** | **Size** | **Conc.** |
| --- | --- | --- | --- | --- | --- | --- | --- | --- | --- |
| **τ** | 1.000 | -0.934 | 0.939 | 0.498 | 0.645 | 0.642 | -0.016 | -0.076 | 0.426 |
| **g** | -0.934 | 1.000 | -0.775 | -0.619 | -0.771 | -0.657 | 0.201 | -0.135 | -0.548 |
| **s** | 0.939 | -0.775 | 1.000 | 0.444 | 0.430 | 0.646 | 0.285 | -0.287 | 0.234 |
| **FAD** | 0.498 | -0.619 | 0.444 | 1.000 | 0.712 | 0.603 | 0.069 | 0.046 | 0.293 |
| **NAD(P)H** | 0.645 | -0.771 | 0.430 | 0.712 | 1.000 | 0.292 | -0.539 | 0.206 | 0.497 |
| **THG** | 0.642 | -0.657 | 0.646 | 0.603 | 0.292 | 1.000 | 0.271 | -0.281 | 0.022 |
| **ORR** | -0.016 | 0.201 | 0.285 | 0.069 | -0.539 | 0.271 | 1.000 | -0.351 | -0.265 |
| **Size** | -0.076 | -0.135 | -0.287 | 0.046 | 0.206 | -0.281 | -0.351 | 1.000 | 0.857 |
| **Conc.** | 0.426 | -0.548 | 0.234 | 0.293 | 0.497 | 0.022 | -0.265 | 0.857 | 1.000 |

**Table S5.** Isolated serum EV linear correlation coefficients.

|  | **τ** | **g** | **s** | **FAD** | **NAD(P)H** | **THG** | **ORR** | **Size** | **Conc.** |
| --- | --- | --- | --- | --- | --- | --- | --- | --- | --- |
| **τ** | 1.000 | -0.886 | -0.286 | -0.261 | -0.044 | 0.677 | -0.596 | -0.652 | -0.539 |
| **g** | -0.886 | 1.000 | 0.651 | 0.021 | -0.187 | -0.791 | 0.705 | 0.237 | 0.092 |
| **s** | -0.286 | 0.651 | 1.000 | -0.304 | -0.335 | -0.408 | 0.384 | -0.454 | -0.557 |
| **FAD** | -0.261 | 0.021 | -0.304 | 1.000 | 0.940 | 0.046 | -0.236 | 0.474 | 0.454 |
| **NAD(P)H** | -0.044 | -0.187 | -0.335 | 0.940 | 1.000 | 0.354 | -0.521 | 0.339 | 0.355 |
| **THG** | 0.677 | -0.791 | -0.408 | 0.046 | 0.354 | 1.000 | -0.912 | -0.144 | -0.066 |
| **ORR** | -0.596 | 0.705 | 0.384 | -0.236 | -0.521 | -0.912 | 1.000 | 0.099 | 0.072 |
| **Size** | -0.652 | 0.237 | -0.454 | 0.474 | 0.339 | -0.144 | 0.099 | 1.000 | 0.949 |
| **Conc.** | -0.539 | 0.092 | -0.557 | 0.454 | 0.355 | -0.066 | 0.072 | 0.949 | 1.000 |

**Table S6.** *Ex vivo* tissue EV linear correlation coefficients.

|  | **FAD** | **NAD(P)H** | **THG** | **ORR** | **Conc.** |
| --- | --- | --- | --- | --- | --- |
| **FAD** | 1.000 | 0.434 | 0.515 | -0.017 | 0.508 |
| **NAD(P)H** | 0.434 | 1.000 | 0.212 | -0.145 | 0.216 |
| **THG** | 0.515 | 0.212 | 1.000 | 0.327 | 0.982 |
| **ORR** | -0.017 | -0.145 | 0.327 | 1.000 | 0.323 |
| **Conc.** | 0.508 | 0.216 | 0.982 | 0.323 | 1.000 |

**Table S7.** Isolated urinary EV vs. *ex vivo* tissue EV linear correlation coefficients.

|  | | ***Ex vivo* tissue** | | | | |
| --- | --- | --- | --- | --- | --- | --- |
|  |  | **FAD** | **NAD(P)H** | **THG** | **ORR** | **Conc.** |
| **Isolated urinary EVs** | **τ** | 0.161 | 0.327 | 0.652 | 0.296 | 0.590 |
|  | **g** | 0.093 | -0.402 | -0.535 | -0.224 | -0.480 |
|  | **s** | 0.325 | 0.226 | 0.634 | 0.365 | 0.548 |
|  | **FAD** | -0.141 | 0.611 | -0.080 | -0.065 | -0.147 |
|  | **NAD(P)H** | 0.036 | 0.798 | 0.244 | -0.088 | 0.259 |
|  | **THG** | -0.450 | -0.132 | 0.026 | 0.398 | -0.048 |
|  | **ORR** | 0.186 | -0.335 | -0.091 | 0.125 | -0.233 |
|  | **Size** | -0.241 | 0.119 | 0.042 | -0.733 | 0.030 |
|  | **Conc.** | -0.030 | 0.302 | 0.365 | -0.590 | 0.319 |

**Table S8.** Isolated serum EV vs. *ex vivo* tissue EV linear correlation coefficients.

|  | | ***Ex vivo* tissue** | | | | |
| --- | --- | --- | --- | --- | --- | --- |
|  |  | **FAD** | **NAD(P)H** | **THG** | **ORR** | **Conc.** |
| **Isolated serum EVs** | **τ** | 0.112 | 0.121 | 0.273 | 0.545 | 0.340 |
|  | **g** | -0.020 | -0.293 | -0.406 | -0.295 | -0.481 |
|  | **s** | 0.000 | -0.211 | -0.392 | 0.365 | -0.486 |
|  | **FAD** | -0.363 | 0.288 | -0.211 | -0.207 | -0.102 |
|  | **NAD(P)H** | -0.251 | 0.543 | -0.190 | -0.155 | -0.082 |
|  | **THG** | 0.021 | 0.635 | 0.051 | 0.086 | 0.086 |
|  | **ORR** | -0.196 | -0.737 | -0.151 | -0.165 | -0.218 |
|  | **Size** | -0.235 | 0.134 | 0.076 | -0.625 | 0.059 |
|  | **Conc.** | -0.175 | 0.245 | 0.219 | -0.618 | 0.199 |

**Table S9.** Isolated urinary EV vs. isolated serum EV linear correlation coefficients.

|  | | **Isolated serum EVs** | | | | | | | | |
| --- | --- | --- | --- | --- | --- | --- | --- | --- | --- | --- |
|  |  | **τ** | **g** | **s** | **FAD** | **NAD(P)H** | **THG** | **ORR** | **Size** | **Conc.** |
| **Isolated urinary EVs** | **τ** | 0.436 | -0.536 | -0.237 | -0.282 | -0.144 | 0.342 | -0.174 | -0.144 | 0.132 |
|  | **g** | -0.327 | 0.535 | 0.316 | 0.011 | -0.118 | -0.426 | 0.263 | -0.088 | -0.338 |
|  | **s** | 0.491 | -0.466 | -0.059 | -0.581 | -0.427 | 0.293 | -0.110 | -0.346 | -0.105 |
|  | **FAD** | 0.291 | -0.422 | -0.100 | -0.086 | 0.183 | 0.785 | -0.565 | 0.023 | 0.137 |
|  | **NAD(P)H** | 0.298 | -0.552 | -0.438 | 0.368 | 0.587 | 0.675 | -0.612 | 0.142 | 0.369 |
|  | **THG** | 0.496 | -0.450 | 0.063 | -0.392 | -0.279 | 0.381 | -0.050 | -0.333 | -0.194 |
|  | **ORR** | -0.100 | 0.365 | 0.611 | -0.880 | -0.874 | -0.224 | 0.378 | -0.320 | -0.404 |
|  | **Size** | -0.629 | 0.206 | -0.520 | 0.455 | 0.322 | -0.147 | 0.159 | 0.974 | 0.972 |
|  | **Conc.** | -0.359 | -0.061 | -0.588 | 0.195 | 0.158 | 0.038 | 0.065 | 0.786 | 0.928 |
